# 3D Cartesian fast interrupted steady‐state (FISS) imaging

**DOI:** 10.1002/mrm.27830

**Published:** 2019-06-14

**Authors:** Thomas Küstner, Aurélien Bustin, Olivier Jaubert, Radhouene Neji, Claudia Prieto, René Botnar

**Affiliations:** ^1^ School of Biomedical Engineering and Imaging Sciences King's College London, St. Thomas’ Hospital London United Kingdom; ^2^ MR Research Collaborations, Siemens Healthcare Limited Frimley United Kingdom; ^3^ Escuela de Ingeniería Pontificia Universidad Católica de Chile Santiago Chile

**Keywords:** 3D Cartesian, bSSFP, cardiovascular imaging, fat suppression, FISS, steady‐state

## Abstract

**Purpose:**

To enable intrinsic and efficient fat suppression in 3D Cartesian fast interrupted steady‐state (FISS) acquisitions.

**Methods:**

A periodic interruption of the balanced steady‐state free precession (bSSFP) readout train (FISS) has been previously proposed for 2D radial imaging. FISS modulates the bSSFP frequency response pattern in terms of shape, width and location of stop band (attenuated transverse magnetization). Depending on the FISS interruption rate, the stop band characteristic can be exploited to suppress the fat spectrum at 3.5 ppm, thus yielding intrinsic fat suppression. For conventional 2D Cartesian sampling, ghosting/aliasing artifacts along phase‐encoding direction have been reported. In this work, we propose to extend FISS to 3D Cartesian imaging and report countermeasures for the previously observed ghosting/aliasing artifacts. Key parameters (dummy prepulses, spatial resolution, and interruption rate) are investigated to optimize fat suppression and image quality. FISS behavior is examined using extended phase graph simulations to recommend parametrizations which are validated in phantom and in vivo measurements on a 1.5T MRI scanner for 3 applications: upper thigh angiography, abdominal imaging, and free‐running 5D CINE.

**Results:**

Using optimized parameters, 3D Cartesian FISS provides homogeneous and consistent fat suppression for all 3 applications. In upper thigh angiography, vessel structures can be recovered in FISS that are obscured in bSSFP. Fat suppression in free‐running cardiac CINE resulted in less fat‐related motion aliasing and yielded better image quality.

**Conclusion:**

3D Cartesian FISS is feasible and offers homogeneous intrinsic fat suppression for selected imaging parameters without the need for dedicated preparation pulses, making it a promising candidate for free‐running fat‐suppressed imaging.

## 1 INTRODUCTION

Balanced steady‐state free precession (bSSFP) is a rapid imaging sequence that provides high signal‐to‐noise ratio (SNR) and excellent blood to muscle contrast without the need of contrast administration. bSSFP is susceptible to off‐resonance effects induced by local B_0_ field inhomogeneities leading to a periodic amplitude modulation of the transverse steady‐state magnetization.^1^ Therefore, the signal response of the bSSFP sequence has pass and stop band regions as a function of the off‐resonance frequency.^2^ In stop band regions, the transverse magnetization drops close to zero, which is perceived as dark bands in the image, so‐called banding artifacts. Several techniques have been proposed to mitigate banding artifacts.^3^, ^4^, ^5^, ^6^, ^7^, ^8^, ^9^

For fat suppression, bSSFP sequences are often combined with frequency selective radiofrequency (RF) pulses which only saturate the fat magnetization^10^ or with inversion recovery pulses and appropriate inversion time to null the signal of the fat magnetization.^11^ This is especially of interest for cardiovascular MRI which requires adequate fat suppression, to image for example coronary arteries that are embedded in epicardial fat.^12^ However, these methods tend to be sensitive to B_0_ or B_1_ field inhomogeneities causing spatial misregistration, uneven fat suppression and/or require additional scan time by the inclusion of the preparation modules.

Therefore, methods have been proposed providing integrated fat suppression of bSSFP imaging. An adapted phase cycling scheme has been proposed to modify the bSSFP frequency response function and broaden the stop band, denoted as fluctuating equilibrium MR (FEMR).^13^ In linear combination SSFP (LC‐SSFP), the modulation of the frequency response function is achieved by acquiring and linearly combining several images with different frequency response patterns. To overcome the long acquisition time limitation of FEMR and LC‐SSFP, the alternating repetition time (ATR)^14^ method was introduced that switches between 2 analytically optimized TR values and a tailored RF phase cycling. Scheffler et al^15^ proposed an alternative magnetization preparation where a fat saturation block is periodically repeated within a continuously running 3D bSSFP sequence. With this approach, the established steady‐state transverse magnetization is stored in the longitudinal component by an α/2 flip‐back pulse before the fat saturation block. Hence, signal contrast and steady‐state of only fat is modified, yielding a fat‐suppressed image. In contrast, Derbyshire et al^16^ proposed S5FP, a spectrally selective suppression with bSSFP. With this approach, a series of 2D Cartesian echo trains is encapsulated by opening (linear ramp‐up RF pulses) and closing (flip‐back pulses with analytically optimized angles) modules, which divide the magnetization into 2 phase‐opposed spectral components A drawback of S5FP is that it requires data scaling during the reconstruction to avoid ghosting/aliasing artifacts in the phase‐encoding direction.

The work of Koktzoglou and Edelman^17^, ^18^, ^19^ continued on these developments by proposing a more frequent interruption (≈ 50‐100 Hz instead of 10‐20 Hz for S5FP) of the bSSFP echo train, which is, therefore, denoted as fast interrupted steady‐state (FISS). FISS uses store and restore *α*/2 pulses encapsulating *n* bSSFP echoes. Depending on the rate of interruption (and by only acquiring a single image compared with: FEMR and LC‐SSFP), this modulates the frequency response of the sequence in a sense that the stop bands are broadened and shifted toward the fat signal at 3.5 ppm. FISS uses a 2D radial trajectory to reduce flow and motion sensitivity and to avoid ghosting artifacts experienced for 2D Cartesian scans along the phase‐encoding direction.

In this work, we sought to build upon the works^16^, ^17^, ^18^ and propose a 3D Cartesian FISS sequence. We focus on the usage of FISS for fat‐suppression which is especially interesting for applications where spectral‐selective fat suppression techniques demand high specific absorption rate and are inefficient due to rapid regrowth of the magnetization or where spectral‐selective excitation prolongs TR and scan time, as it is the case for free‐running (continuous acquisition under free‐breathing with retrospective cardiac gating when needed) acquisitions,^20^ peripheral angiography,^21^ and cardiac MRI.^12^ Our contributions include (i) the extension of FISS toward 3D Cartesian k‐space sampling which can be integrated into any 3D bSSFP sequence, (ii) the analysis of imaging parameters and associated pass and stop bands (frequency response) using extended phase graph (EPG) simulations to identify the optimal operating range for fat suppression and best image quality. The proposed 3D Cartesian FISS sequence is validated in a phantom experiment and in vivo in 18 healthy subjects for 3D upper thigh angiography, abdominal MRI and free‐running 5D (3D cardiac and respiratory resolved) cardiac CINE.

## 2 METHODS

The frequent interruption of the bSSFP train of FISS modulates the frequency response pattern.^17^ The rate of interruption 1/TRf determines the width, location and shape of the stop band which is used for fat suppression, with TRf being the overall FISS module duration. The fat suppression capability depends on the number *n* of bSSFP readouts per FISS module, echo time (TE)/TR, bandwidth and duration of dephasing gradients which contribute to TRf. A given desired imaging resolution (along the frequency encoding direction) determines, for a given bandwidth, the TE/TR and thereby the timing in and between the FISS modules (for a fixed dephasing duration). Thus, bandwidth and/or the number *n* of bSSFP readouts per FISS module can be modified to guarantee that the stop band fall into the fat spectrum range (3.5 ppm ≡ ±220 Hz for 1.5T) for a given resolution. In applications with minimal TE/TR (i.e., maximal bandwidth) and given desired imaging resolution such as free‐running cardiac CINE, the choice of the optimal number *n* determines the degree of fat suppression.

We first propose a generic 3D Cartesian FISS sequence and then analyze the optimal operating range according to MR sequence parameters as well as the inter‐dependency of these parameters. Sequence parameters are analyzed based on EPG simulations and subsequently validated in phantom and in vivo measurements.

To avoid the previously reported ghosting/aliasing artifact along the phase‐encoding direction,^16^, ^17^ several key parameters of the 3D Cartesian FISS were examined including: (a) RF phase cycling and gradient spoiling, (b) dummy preparation pulses, and (c) FISS interruption rate, flip angle.

### 2.1 3D Cartesian FISS sequence

**Figure 1:**
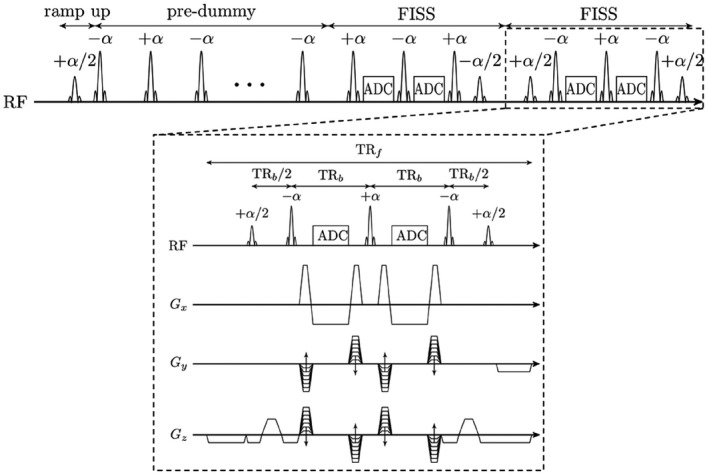
3D Cartesian FISS pulse sequence diagram showing the continuous acquisition train with dummy prepulses followed by FISS modules with *α*/2 RF ramp‐up/down pulses. One FISS module (*n* = 2 here) consists of [+ramp‐up, −*α*, ADC, +*α*, ADC, −*α*, + ramp‐down] with flip angle *α* and receiver ADC. Phase‐encoding gradients *G_y_*, *G_z_* are rewinded and RF phases are cycled between FISS modules. The moment of the frequency gradient *G_x_* is nulled. TRf describes the duration of the FISS module (including gradient spoiling) and TRb the repetition time between bSSFP readouts

The basic pulse sequence of the 3D Cartesian FISS is shown in Figure 1. A FISS module is composed of an α/2 ramp‐up pulse, a series of *n* bSSFP readouts with phase‐alternating ±α RF pulses and an α/2 store pulse.^15^, ^16^ Gradient moment nulling is fulfilled for the frequency encoding direction. Rewinder gradients are applied along the phase‐encoding directions. 3D Cartesian FISS is combined with a variable‐density spiral‐like Cartesian (VD‐CASPR) sampling trajectory.^22^ A golden angle increment between consecutive spiral‐like arms ensures optimal k‐space coverage. The trajectory allows undersampling the phase‐encoding plane with given acceleration *R* and enables an efficient sampling scheme for continuous free‐running acquisitions.^23^ However, it shall be noted that 3D Cartesian FISS is independent of the applied Cartesian trajectory and does not require any undersampling.

While Derbyshire et al^16^ used linear ramp‐up pulses^24^ to provide smooth transition and to avoid undesirable transient behavior from isochromats in the off‐resonant region, Koktzoglou and Edelman (17) proposed an α/2 ramp‐up pulse (see Figure 1). Based on a preliminary study (see Supporting Information Text S1, which is available online), we focus on the α/2 pulses.

#### 2.1.1 RF phase cycling and gradient spoiling

Dephasing between FISS modules is achieved by the combination of RF and gradient spoiling, reducing signal from residual transverse magnetization and resulting in a broader stop band characteristic. The duration *t*_spoil_ of the spoiling gradients strongly determines the rate of interruption 1/TRf with

(1) $$\text{TRf}\;=\left(n+1\right)\;\cdot \;\text{TRb}\;+\;t_{\text{spoil}}$$

where TRb describes the echo spacing between readouts. The RF phase cycling uses a phase increment *φ_j_* at the *j*‐th FISS module according to

(2) $$\varphi_{j}=\varphi_{j-1}+j\cdot \varphi_{0}$$

with an initial golden phase *φ*_0_ = 111.3°. This phase cycle scheme ensures sufficient RF spoiling and the initial golden phase avoids coinciding short‐term phases, i.e., short‐term transverse magnetization accumulations.

#### 2.1.2 Dummy preparation pulses

A train of dummy ±α RF preparation pulses with TRb repetition brings the bSSFP magnetization toward a steady‐state. Frequency and phase‐encoding gradient moments are nulled. The FISS interruption and acquisition starts once this steady‐state is reached to avoid off‐resonant transient signal oscillations. These oscillations result in the previously reported ghosting/aliasing artifacts along the phase‐encoding direction for Cartesian sampling.^16^, ^17^ Instead of smoothing out the artifacts by using radial sampling^17^ or slowly ramping up the magnetization,^16^ we propose the usage of a train of dummy prepulses. The train of dummy prepulses consists of *D* RF pulses and needs to be applied only once at the beginning of the acquisition.

### 2.2 EPG simulations

The signal evolution over time and in the frequency domain are simulated using EPG simulations^25^ for different acquisition parameters. A total of 1000 FISS modules are simulated and the frequency profile of the 600th FISS module serves as the steady‐state time point. Transient behavior to the steady‐state is exemplary examined in the 50th FISS module. Frequency response of the off‐resonance signal is determined for a range of precession frequencies from ‐400 Hz to 400 Hz in 1‐Hz increments. The MR imaging parameters are as follows: TE/TRb ∈ [1.69/3.38, 1.53/3.06, 1.47/2.93, 1.43/2.86, 1.39/2.77] ms corresponding to an isotropic imaging resolution of [1.5, 1.8, 2.1, 2.3, 2.7] mm^3^ for maximal readout bandwidth on the scanner (1.5T MRI, Siemens MAGNETOM Aera); flip angle = 50°; size of FISS module n=1,…,5,10,15,20,25,30. Simulations are conducted for fat, liver, myocardium, and blood with respective T1/T2 values of 250/70 ms, 500/40 ms, 1000/45 ms, and 1600/250 ms.

The influences of the timing parameters TE/TRb and *n* (for a fixed spoiling duration *t*_spoil_ = 2.5 ms) are examined for its effectiveness of fat suppression. A broad stop band in the frequency response at around 3.5 ppm ≡ ±220 Hz (for 1.5T) is desired to suppress the broad fat spectrum, i.e., signal cancellation of fat with tolerance to the different ^1^H nuclei in fat molecules. On the other hand, the pass band around on‐resonance should not be affected, to avoid suppression of water signal. All simulations were performed in MATLAB (version R2017a; MathWorks Inc., Natick, MA).

### 2.3 Overall study design

3D Cartesian FISS was tested in phantom and healthy human subjects in upper thigh (*N* = 10, 4 females, age = 29 ± 3 years), abdomen (*N* = 5, 2 females, age = 28 ± 3 years) and heart (*N* = 3, all male, age = 29 ± 2 years). All subjects gave written informed consent and the study was approved by the local ethics committee. Imaging was performed on a 1.5T MRI scanner (MAGNETOM Aera, Siemens Healthcare, Erlangen, Germany). The proposed 3D Cartesian FISS was compared against a 3D Cartesian bSSFP sequence and results were compared with the theoretical findings of the EPG simulations. Timing influence on fat suppression capability and artifact appearance were investigated. The impact of RF preparation pulses and the dummy prepulses was examined in phantom and in vivo imaging for upper thigh. Abdominal and cardiac acquisitions were performed with the optimized imaging parameters. The minimal possible TRb was chosen for all acquisitions to enable efficient and fast imaging.

#### 2.3.1 Phantom study

An oil phantom (T1/T2 = 250/70 ms) was acquired with FISS and compared with bSSFP with and without a spectral attenuated inversion recovery fat saturation (SPIR) pulse,^10^ as well as to a gradient echo (GRE) sequence without fat suppression. The SPIR pulse with a flip angle of 300° is played out every 110 ms (empirical value, not optimized). Acquisition parameters of FISS, bSSFP, and GRE were identical when possible and given by: field of view (FOV) = 300 × 300 × 40‐86 mm (left/right × anterior/posterior × superior/inferior), *α* = 50° (bSSFP and FISS) / 10° (GRE), bandwidth = 1500 Hz/px (FISS) / 1020 Hz/px (bSSFP and GRE), acceleration *R* = 1. Same TE/TRb, *n* values and isotropic resolutions were chosen as in the EPG simulations. The dependency on (i) TRf and TRb, for a fixed spoiling (RF spoiling, gradient spoiling) and *n*, was examined. Moreover, the influence of (ii) RF phase cycling with gradient spoiling, and (iii) dummy preparation pulses was investigated for a fixed resolution of 1.8 mm^3^. Average signal intensity in a volume of interest (VOI) inside the oil phantom (images are scaled to a range of 0 to 1) was examined.

#### 2.3.2 In vivo study

Healthy subjects were scanned with 3D Cartesian FISS and bSFFP (without fat suppression) for upper thigh angiography, breath‐hold abdominal MRI and free‐running cardiac CINE. Transverse upper thigh imaging parameters were the same as in the phantom study except for: FOV = 370 × 205 × 94 mm. The impact of (i) dummy prepulses *D* ∈ [0, 200] for a fixed isotropic resolution of 1.8 mm^3^ and FISS module sizes of *n* ∈ [1, 2, 3, 4, 10], and (ii) imaging resolutions = [1.5, 1.8, 2.1, 2.7] mm^3^ isotropic corresponding to TE/TRb ∈ [1.69/3.38, 1.53/3.06, 1.47/2.93, 1.39/2.77] ms for *n* ∈ [1, 2, 4] with *D* = 200 were examined. SNR in muscle (soleus) and fat (subcutaneous adipose tissue) as well as contrast between these 2 compartments and femoral artery were measured in a drawn VOI in upper thigh images. Reported values are average and standard deviation over all 10 subjects. For the 1.8 mm^3^ resolution upper thigh exam, acquisition times of 19 s (bSSFP), 1:12 / 0:46 / 0:36 / 0:32 / 0:23 min:s (FISS for *n* = 1, 2, 3, 4, 10) were achieved.

Subsequently a transverse breath‐hold abdominal imaging was carried out with same parameters than in the phantom study except for FOV = 370 × 240 × 47 mm, 1.8 mm^3^ isotropic resolution, *n* ∈ [1, 2, 3, 4] corresponding to acquisition times of 10 s (bSSFP), 33 / 21 / 17 / 15 s (FISS for *n* = [1, 2, 3, 4]).

The proposed fat‐suppressed Cartesian FISS was further evaluated in a respiratory self‐navigated (1D self‐navigation: k‐space central line) free‐running 3D whole heart cardiac scan^23^ with: retrospective ECG‐synchronization, coronal orientation, FOV = 360 × 546 × 292 mm (superior/inferior × left/right × anterior/posterior), 1.4 mm^3^ isotropic resolution, TE = 1.59 ms, TRb = 3.1 ms, *α* = 60°, readouts per spiral‐like arm = 30, acceleration *R* = 3, bandwidth = 1230 Hz/px (FISS) / 880 Hz/px (bSSFP), FISS *n* = 2, respiratory self‐navigation period = 110 ms (interval between repeated central k‐space line), acquisition time = 7:52 min:s (for both bSSFP and FISS). Images were retrospectively binned into 4 respiratory and 8 cardiac states based on the respiratory self‐navigator and ECG signal, leading to a 5D cardiac dataset. To deal with the undersampling (after data binning), a patch‐based PROST reconstruction^26^ was used to obtain the cardiac and respiratory resolved images. Reconstruction parameters are described in Bustin et al.^26^

## 3 RESULTS

### 3.1 EPG simulations

**Figure 2:**
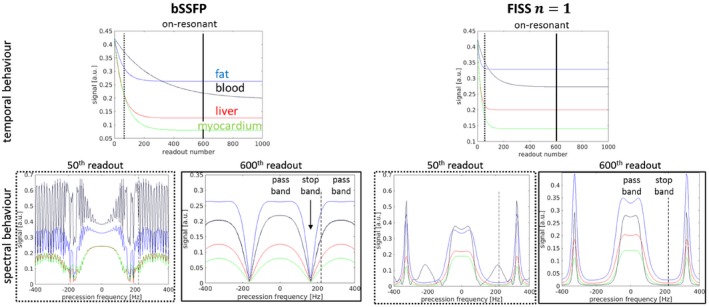
EPG simulations showing the temporal and spectral behavior of bSSFP and FISS *n* = 1 (*α*/2 RF, gradient and RF spoiling) acquisitions for fat (blue), blood (black), liver (red), and myocardial (green) tissue. Simulation parameters for both bSSFP and FISS are: resolution = 1.8 mm^3^, TRb = 3.06 ms, TRf = 8.62 ms, flip angle = 50°. The fat tissue spectrum lies around 3.5 ppm ≡ ±220 Hz for 1.5T (dashed vertical line only shown for +220 Hz). Spectral frequency response is depicted for the 50th (before reaching the steady‐state) and the 600th (after having reached the steady‐state) readout. Vertical bars in temporal on‐resonant signal (at 0 Hz) mark these time points. The stop band in FISS at around 3.5 ppm ensures the fat suppression

EPG simulations for FISS in comparison to bSSFP are shown in Figure 2. The on‐resonant signal through time is depicted to examine the steady‐state behavior and influence of FISS interruption on water signal. The off‐resonant fat signal at 3.5 ppm shows a stronger attenuation in the spectral domain for FISS compared with bSSFP. The frequency response consists of alternating pass and stop bands (symmetric around 0 Hz). The periodic interruption of FISS for the conducted experiments was between 9 Hz (*n* = 30) and 85 Hz (*n* = 1). Stop band width decreases with *n* and increasing resolution, respectively, increasing TRb (see also EPG simulations in Figure 6). A steady‐state was reached after 100‐200 readouts in both bSSFP and FISS. Transition to steady‐state depends on the timing parameters (TRb and n). Faster transition was observed for larger *n* (see Supporting Information Figure S1) and steady‐state in all tissues was reached faster than for bSSFP. EPG simulations indicate that a train duration of ~ 550‐680 ms, corresponding to *D* ≈ 200 (depending on TRb), is sufficient to bring long T1 tissues (≲1000 ms) to steady‐state (for all *n*). Simulations also show that for increasing *n* > 3, the number of predummy pulses could even be decreased to *D* < 200. The transient behavior to steady‐state in the spectral domain is depicted in Figure 2 for the 50th readout. Simulations show that the predummy pulses ensure ghosting/aliasing artifact‐free acquisition after reaching the steady‐state.

**Figure 3:**
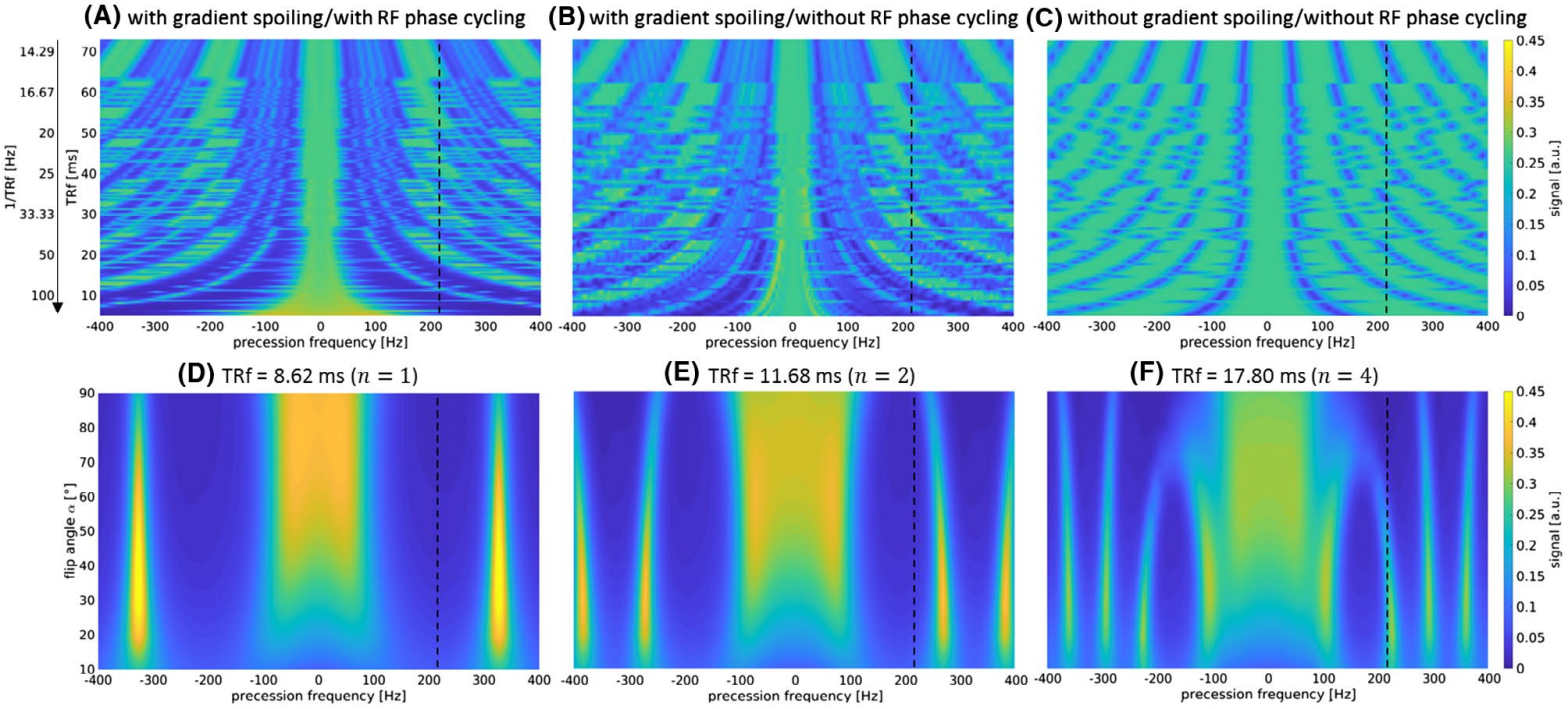
EPG simulations showing the spectral behavior of FISS as a function of interruption rate 1/TRf (at flip angle *α* = 50°, t_spoil_ = 2.5 ms) for gradient spoiling and RF phase cycling (A), gradient spoiling and no RF phase cycling (B), no gradient spoiling and no RF phase cycling (C) and as a function of the flip angle *α* (with gradient spoiling and RF phase cycling) at TRf = 8.62 ms (*n* = 1) (D), TRf = 11.68 ms (*n* = 2) (E), and TRf = 17.80 ms (*n* = 4) (F). Simulations depict the fat tissue (T1 = 250 ms, T2 = 70 ms); blue line in Figure 2. Similar spectral behavior was obtained for the other tissues. The fat spectrum (3.5 ppm ≡ ±220 Hz for 1.5T) is marked by the vertical dashed line

The influence on the frequency response pattern of fat (blue line in Figure 2) for changing interruption rates 1/TRf (with and without gradient/RF spoiling) and flip angles *α* is shown in Figure 3. Gradient spoiling and RF phase cycling provide a sufficiently broad stop band for fat suppression for an interruption rate of 1/TRf ≳ 50 Hz. A flip angle of >30° ensures sufficient on‐resonant water signal.

### 3.2 Phantom study

**Figure 4:**
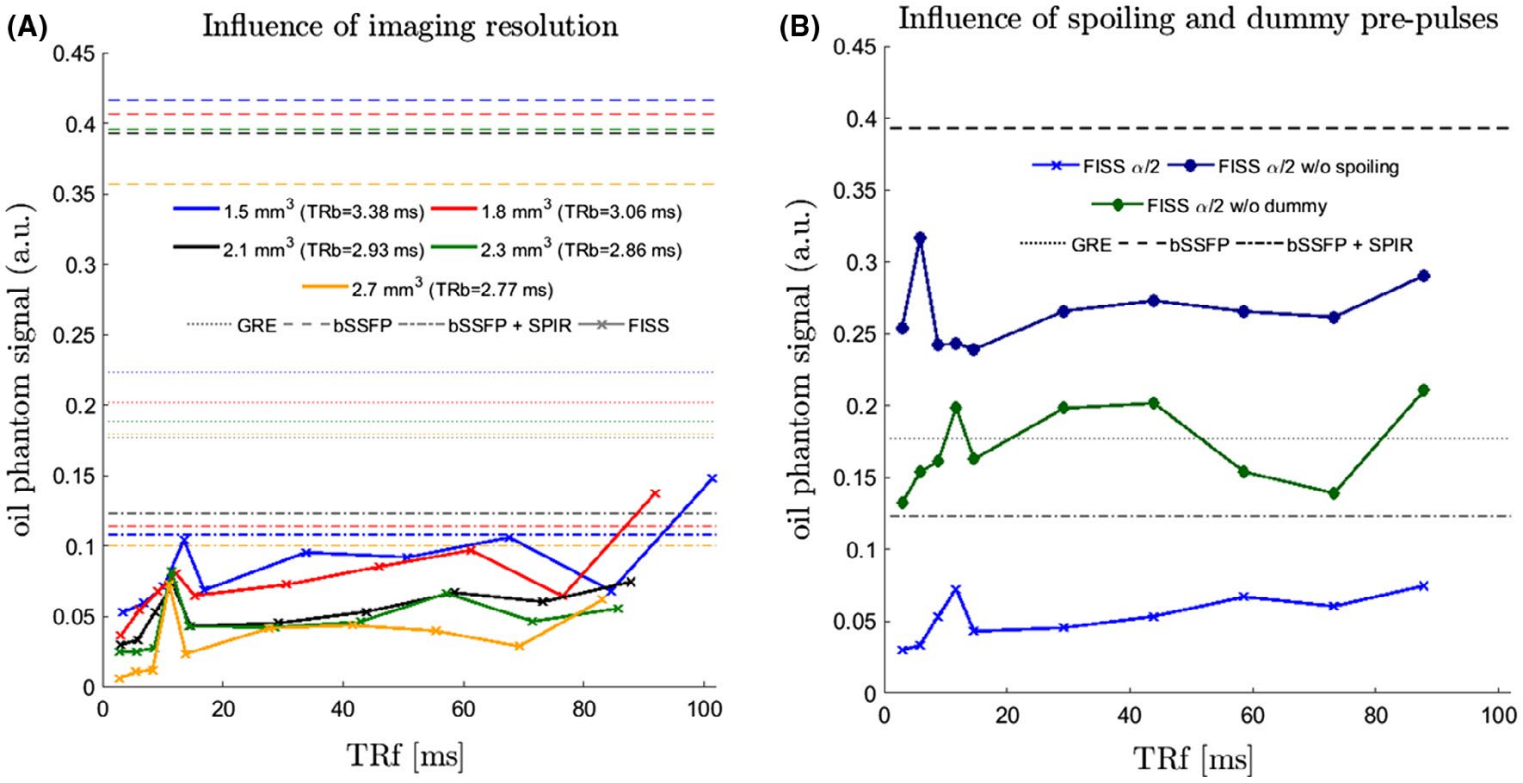
Phantom measurements to examine the influence of imaging resolution, respectively TRb (A), gradient spoiling and dummy prepulses over repetition time TRf (B). Lines show the respective average signal intensity measured in a VOI of an oil phantom which has been imaged with bSSFP (dashed lines), bSSFP with SPIR fat saturation (dash‐dotted lines), GRE (dotted lines), and FISS (continuous lines). Each point along the FISS lines corresponds to a FISS module number n = 1, 2, 3, 4, 5, 10, 15, 20, 25, 30 (from left to right)

The phantom experiments show the influence of *n* and TRf for varying imaging resolution (Figure 4A), gradient spoiling with phase cycling and predummy pulses (Figure 4B) on fat suppression effectiveness. The VOI analysis reflects the fat suppression effectiveness and image quality (artifact influence). Fat signal in FISS is always lower than the corresponding bSSFP measurement. Following signal evolution in Figure 4 (the lower the signal intensity of oil/fat the better), shorter TRf (smaller *n*) should be favored for most effective fat suppression. For the examined timing parameters, EPG simulations suggested a reduced fat suppression efficiency starting from *n* = 4, i.e., from 1/TRf ≲ 50 Hz, which was verified by the phantom measurements.

Similar fat suppression was observed for all examined spatial resolutions with an average deviation among them of < 3%. Fat suppression was superior in FISS for all resolutions and *n* ≤ 25 compared with bSSFP with SPIR and GRE without SPIR.

**Figure 5:**
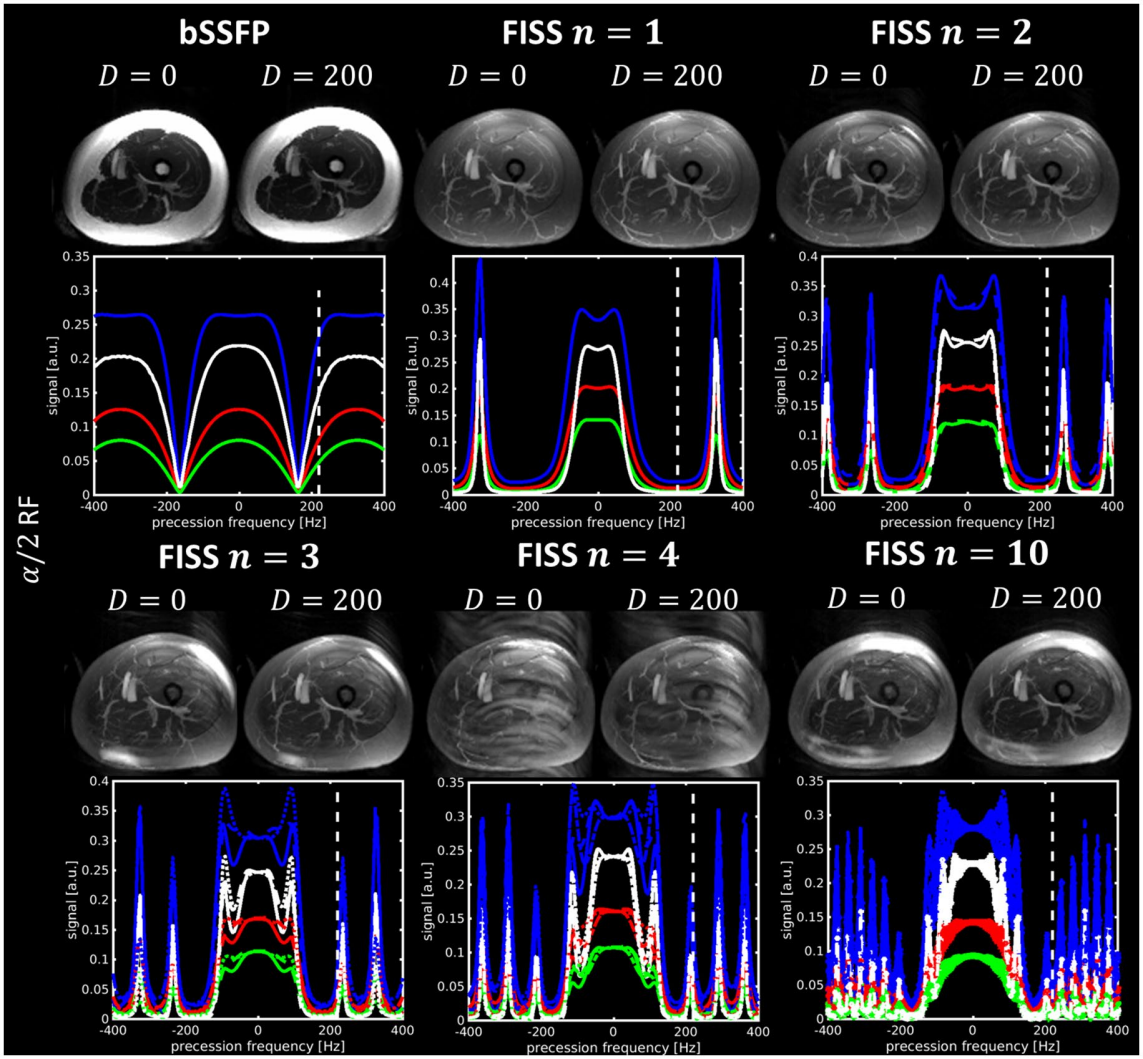
Transverse MIPs in upper thigh for bSSFP and FISS acquisitions (*n* = 1, 2, 3, 4, 10) with *α*/2 RF pulse and with (*D* = 200) and without (*D* = 0) dummy prepulses acquired with 1.8 mm^3^ isotropic resolution (TRb = 3.06 ms, TRf = 8.62 ms). The spectral behavior depicts the frequency response of fat (blue), blood (black), liver (red), and myocardial (green). Lines with the same color and different line style (for FISS *n* > 2) show the profiles of each individual bSSFP readout. Dashed vertical line indicates the positive resonance frequency of fat tissue (3.5 ppm ≡ ±220 Hz for 1.5T)

Gradient spoiling and RF phase cycling strongly improved fat suppression (see also Koktzoglou and Edelman).^17^ Without gradient spoiling and RF phase cycling, suppression was nearly not visible and mean signal intensity was even higher than in bSSFP. RF phase cycling and gradient spoiling widen and increase attenuation of the stop band with a sharper transition to the on‐resonant pass band as shown in Supporting Information Figure S2. Without gradient spoiling between FISS modules, no stop band was achieved and stronger oscillations occurred in the pass band around the on‐resonance signal. However, gradient spoiling did not influence the on‐resonant water signal amplitude. Empirically a minimal gradient moment of 2.6751*π* per mm was determined. Besides spoiling, the second strongest improvement arises from the dummy prepulses suppressing the ghosting/aliasing appearance which is reflected in a smoother and consistent signal in the VOI (see Figure 5).

### 3.3 In vivo study

Parameter selection optimized with EPG simulations was verified in vivo in upper thigh angiography. Figure 5 depicts the maximum intensity projection (MIP) along axial direction together with EPG simulated frequency responses in FISS steady‐state to illustrate the influence of dummy prepulses. Fat suppression was excellent and homogeneous over the whole volume for FISS while absent in bSSFP without fat suppression. Ghosting/aliasing artifacts were only observed for FISS *n* = 4, because of the narrow pass band at 3.5 ppm. Homogeneity and strength of fat suppression decreased for FISS with increasing module number *n*. Dummy prepulses mitigated the occurrence of ghosting/aliasing artifacts.

**Figure 6:**
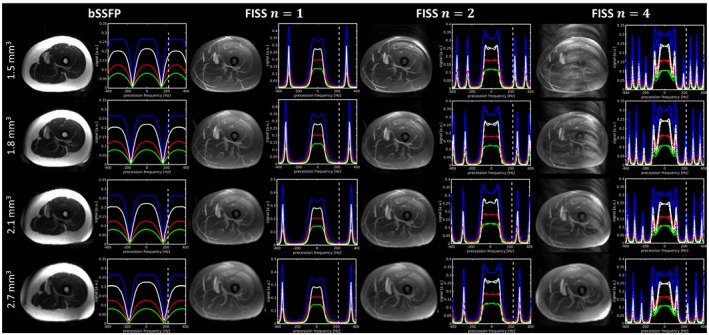
Transverse MIPs in upper thigh for bSSFP and FISS acquisitions (*n* = 1, 2, 4) with *α*/2 RF and *D* = 200 dummy prepulses, t_spoil_ = 2.5 ms and for varying repetition time TRb (3.38 ms, 3.06 ms, 2.93 ms, 2.77 ms), respectively, imaging resolution (1.5 mm^3^, 1.8 mm^3^, 2.1 mm^3^, 2.7 mm^3^). The spectral behavior depicts the frequency response of fat (blue), blood (black), liver (red), and myocardial (green). Lines with the same color and different line style (for FISS *n* > 2) show the profiles of each individual bSSFP readout. Dashed vertical line indicates the positive resonance frequency of fat tissue (3.5 ppm ≡ ±220 Hz for 1.5T)

Lower resolutions can provide more robust fat suppression as shown in Figure 6. For increasing imaging resolution and FISS module number *n*, respectively, increased TRf, the stop band got narrower and the neighboring pass band moved into the frequency range of the fat signal (at 3.5 ppm). For a resolution of 1.8 mm^3^, the stop band spans from ±0.35/TRb to ±0.90/TRb for *n* = 1 and reduces to a width of ±0.35/TRb to ±0.65/TRb for *n* = 3. For large *n*, the stop band narrowed little with increasing resolution while it narrowed more significantly for smaller *n*. The stop band narrowed rapidly between *n* = 1 to *n* = 4 after which the first off‐resonant pass band was at ≤ 3.5 ppm (depending on TRb). As long as the first off‐resonant pass band was ≥ 3.5 ppm, no ghosting/aliasing artifacts were observed in the MIP images (see changing resolution/TRb for FISS *n* = 4 in Figure 6). Fat signal attenuation (off‐resonant fat signal FISS to bSSFP) decreased on average over all examined timings linearly from ‐10 dB to ‐4 dB from FISS *n* = 1 to *n* = 4 and stays afterward for *n* > 4 fairly constant at ‐4 dB.

**Figure 7:**
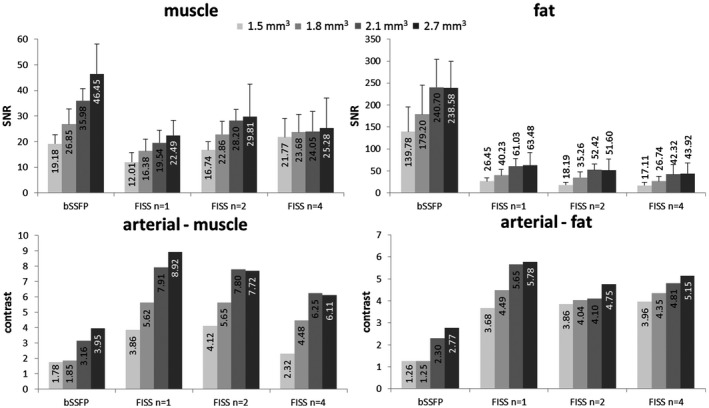
Quantitative analysis of VOIs drawn in soleus muscle, subcutaneous adipose tissue and femoral artery of upper thigh imaging (same acquisition protocol as in Figure 6). Bar plots represent the average over all subjects. Standard deviations for contrast are negligibly small

A quantitative analysis including SNR and contrast is shown in Figure 7. SNR in the muscle was higher in bSSFP than in FISS due to the gradient and RF spoiling used in FISS. Highest percentage decrease of FISS *n* = 1 to bSSFP ranged from 37% (1.5 mm^3^) to 52% (2.7 mm^3^). With decreasing resolution and increasing *n*, SNR increased in muscle. SNR in fat was lower for FISS than for bSSFP with an average decrease of fat SNR of 81%, demonstrating its good fat suppression. SNR in fat for FISS *n* = 4 is lower than for FISS with *n* = 1, because of signal voids arising from the ghosting/aliasing artifacts. The SNR in muscle remains fairly constant for FISS *n* = 4 with different resolutions, which could be explained by the existence of aliasing signal. Arterial‐to‐muscle contrast was improved by 125% on average and arterial‐to‐fat contrast by 162% for FISS compared with bSSFP. Largest improvements were achieved for lower *n*.

**Figure 8:**
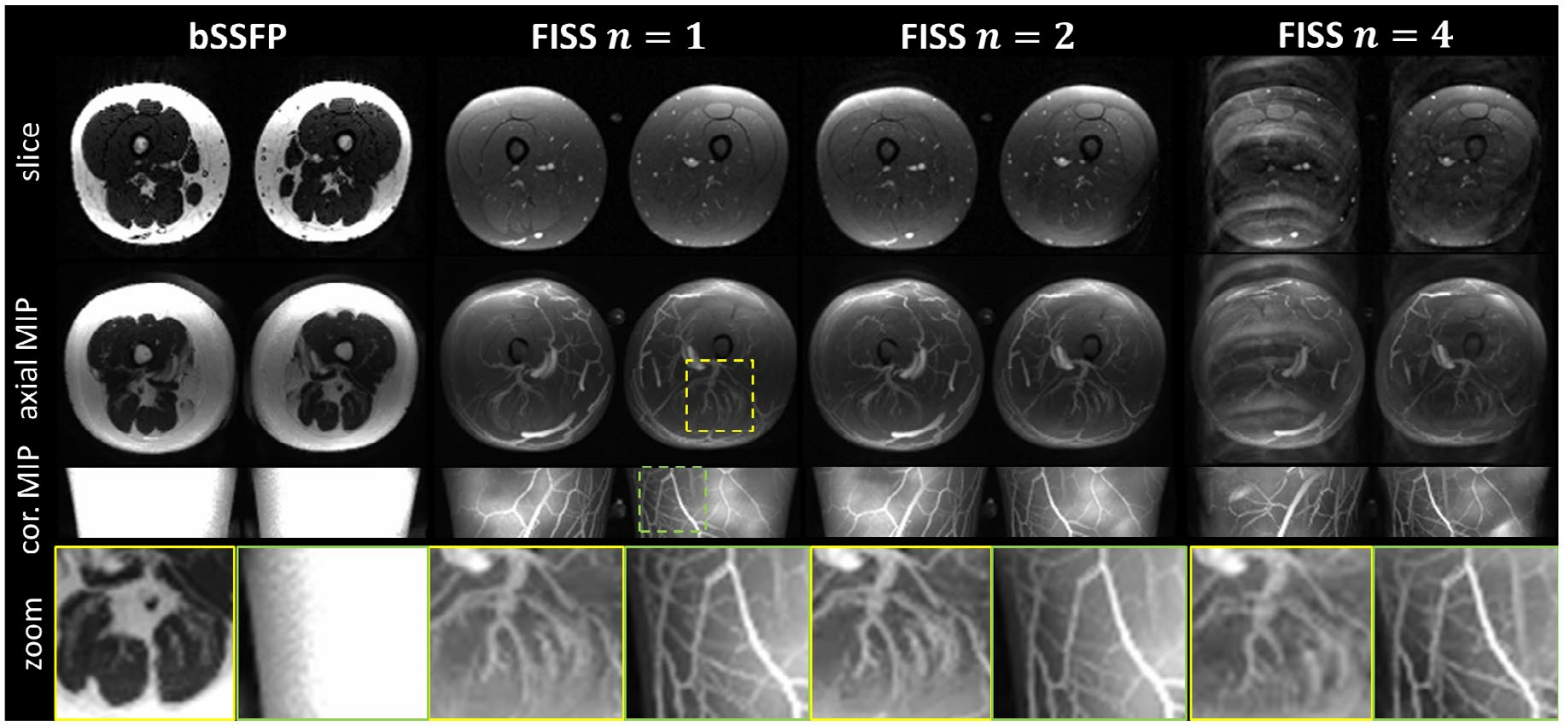
Representative slice and axial/coronal MIP for bSSFP and FISS acquisitions (*n* = 1, 2, 4, *α*/2 RF, *D* = 200) in upper thigh of a subject with a slightly elevated BMI of 28 kg/m^2^. Vessel depiction is possible with FISS and impaired in bSSFP

A representative slice and axial and coronal MIPs are shown for 2 subjects with varying body mass index (BMI) and subcutaneous adipose tissue volume in Figure 8 (BMI = 28 kg/m^2^) and Supporting Information Figure S3 (BMI = 20 kg/m^2^). Fat suppression was excellent and homogeneous over the whole volume for FISS and enabled depiction of small vessels and structures which were lost with bSSFP without fat suppression. Based on the T2/T1 weighting nature of FISS, the venous blood signal is assumed to be lower (lower blood oxygenation and, hence, shorter T2) and, therefore, mainly arteries are depicted. Contrast between muscle and vessel and fat and vessel were also improved. The presence of ghosting/aliasing artifact in FISS with *n* = 4 was more prominent in subjects with larger subcutaneous adipose tissue volume and predominantly occurred along the *k_y_* phase encoding direction. Coronal MIPs still provide acceptable vessel visualization, however, some ghosting/aliasing artifacts prevent clear vessel delineation. Presence of ghosting/aliasing artifacts is also due to B_0_ inhomogeneity as observed in Supporting Information Figure S3 for lateral subcutaneous adipose tissue in the right thigh.

**Figure 9:**
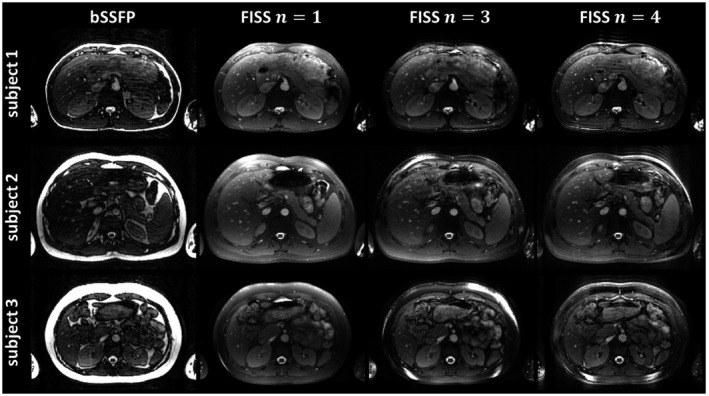
Representative bSSFP and FISS abdominal slices (*n* = 1, 3, 4, *α*/2 RF, *D* = 200) acquired under breath‐hold for 3 subjects with low (subject 1) and high (subjects 2 and 3) BMIs. FISS fat suppression is excellent and consistent over the whole volume, but susceptible to B_0_ inhomogeneity

In Figure 9, the application of FISS to abdominal breath‐hold imaging is shown in 3 different subjects with varying subcutaneous adipose tissue volume. Subcutaneous adipose tissue and visceral adipose tissue were homogeneously suppressed. Susceptibility to B_0_ inhomogeneities can be observed in the superficial subcutaneous adipose tissue for FISS *n* ≥ 3. Ghosting/aliasing artifacts were not observed for FISS *n* = 4. As shown in Supporting Information Figure S4 for upper thigh and abdominal imaging, 3D Cartesian FISS is independent of the applied Cartesian sampling trajectory.

**Figure 10:**
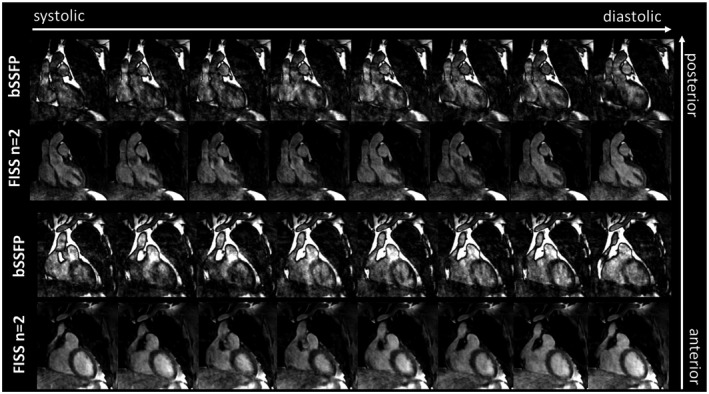
Cardiac motion‐resolved images at end‐expiration of a free‐running cardiac CINE acquisition at 1.4 mm^3^ isotropic resolution with bSSFP and FISS (*n* = 2, *α*/2 RF, *D* = 200) over 2 exemplary slices. Whole‐heart coverage over all slices is shown in Supporting Information Video S1. Data were retrospectively binned into 4 respiratory and 8 cardiac phases from an acquisition of 7:52 min:s duration (with an acceleration factor *R* = 3 for FISS), i.e., roughly twice the amount of data is available for bSSFP in comparison to FISS. Fat‐related motion aliasing can be reduced with FISS improving the image quality despite the higher undersampling factor in comparison to bSSFP

A free‐running cardiac CINE imaging case is shown in Figure 10 and whole‐heart coverage in Supporting Information Video S1 for bSSFP without fat suppression and FISS *n* = 2. Note that the same acquisition time was used, i.e., for FISS (due to the periodic interruption) only half the amount of data was acquired compared with bSSFP. Due to the improved fat suppression of FISS fat‐related artifacts originating from moving epicardial and pericardial fat due to respiratory and cardiac motion have been reduced despite the higher undersampling factor. Fat suppression was homogeneous across the FOV and motion states.

Results from all experiments suggest that a reliable operating range for Cartesian imaging is given by small FISS module numbers *n* < 4 with minimal TE/TRb for an acquisition with *α*/2 RF pulses, gradient spoiling, RF phase cycling with golden angle and dummy prepulses *D* ≈ 100 – 200.

## 4 DISCUSSION

In this work, we proposed the extension of FISS for 3D Cartesian imaging and optimized key sequence parameters in simulations, phantoms and in vivo studies. For 3D Cartesian FISS, the main findings were (1) fat suppression efficiency depends on the periodic FISS interruption rate and by that on the imaging parameters; (2) predummy pulses, RF phase cycling, and gradient spoiling enable 3D Cartesian FISS; and (3) the occurrence of the aliasing/ghosting artifact arises from pass band characteristic at 3.5 ppm.

The periodic interruption of the bSSFP readout train by RF ramp‐up and ramp‐down pulses modulates the frequency response pattern and can create a desired stop band at 3.5 ppm. The width, shape and attenuation depend on the imaging parameters, mainly TE/TRb, bandwidth, and number of bSSFP readouts per FISS module *n*, which contribute to the interruption rate 1/TRf. EPG simulations revealed the best operating range for several imaging parameters and the simulation results were then verified by phantom measurements and in vivo imaging.

In general, a homogeneous fat suppression over the whole FOV was achieved without the need of explicit fat saturation pulses. We identified a reliable operating range for Cartesian imaging for *n* < 4 (minimal TE/TRb), *α*/2 RF pulses, gradient spoiling, RF phase cycling with golden angle and dummy prepulses *D* ≈ 100 – 200. In contrast to Edelman et al^18^ who reported a FISS module number *n* up to 8 with radial k‐space sampling, we found reliable operation of *n* < 4 among all examined applications. However, we also observed good fat suppression in the phantom for larger FISS module numbers *n* = 20 – 25, which is supported by the EPG simulation experiments for changing TRf (Figure 3). This needs further evaluation in vivo and could be interesting for applications with longer scan times, such as free‐running cardiac imaging. Fat suppression in FISS depends on the timing parameters (interruption rate) which need to be optimized for each application. In general, we concur with the proposed FISS interruption rate in Koktzoglou and Edelman^17^ of 1/TRf ≥ 50 Hz.

However, in some applications, there may be other optimal parametrizations. For example, in abdominal imaging FISS *n* = 4 produced satisfactory results while ghosting/aliasing artifacts were present for upper thigh angiography with *n* = 4. The reason for this difference may stem from the composition of the fat: subcutaneous adipose tissue and visceral adipose tissue are present in the abdomen whereas mainly subcutaneous adipose tissue and oelefinic fat (at around +0.5 ppm) is present in the upper thigh. In contrast to phantom measurements where the fat peaks have a narrow linewidth, in vivo measurements get worse for larger *n*, because several adipose tissues contribute to a broader fat spectrum which becomes more difficult to suppress (depending also on the B_0_ homogeneity across the FOV).

SNR and contrast measurements may not fully capture the human visual perception of the images. However, it can provide sufficient indication if the proposed fat suppression is working and if the water signal is affected. The reported SNR and contrast improvements are in agreement with the observed changes in 2D radial FISS in Koktzoglou and Edelman,^17^ i.e., 3D Cartesian FISS enables similar fat suppression. Similar dependency of fat suppression on FISS module number *n* was observed in the work of Koktzoglou and Edelman.^17^ They concluded that radial imaging is less sensitive to signal fluctuations than Cartesian for *n* > 1 which is supported by the findings in this work. However, 3D Cartesian FISS is feasible if operated with the proposed modifications and within determined operating ranges.

FISS is less sensitive to flow artifacts from off‐resonant and out‐of‐volume spins, due to periodic gradient and RF spoiling. These conclusions have been previously reported in Koktzoglou and Edelman^17^ and have been qualitatively confirmed for free‐running 5D Cartesian FISS (3D Cartesian, cardiac and respiratory resolved), but requiring more detailed analysis in future studies.

The direct use of Cartesian sampling for FISS amplified the severity of the ghosting/aliasing artifacts for certain timing and parameter settings which was not observed for radial trajectories because artifacts are more uniformly distributed over the entire FOV.^17^ The described extensions help to minimize these artifacts. A VD‐CASPR sampling scheme with a spiral‐like trajectory was used, but similar results were obtained with conventional Cartesian sampling with and without parallel imaging rendering the method independent to the Cartesian reordering scheme. Our results show that the occurrence of the ghosting/aliasing artifact in Cartesian imaging can be avoided if the stop band is broad enough at around 3.5 ppm and if sufficient dummy prepulses are applied, i.e., imaging starts after the FISS steady‐state is reached. A large number *D* = 200 was selected, but EPG simulations indicate that a shorter train (~100‐200) could be sufficient.

Koktzoglou and Edelman^17^ proposed a flip angle *α* > 30°. Following the EPG simulations in Figure 3, a satisfactory on‐resonant pass band is obtained for *α* > 30°. Consequently, we chose a maximal flip angle in the range of 50° to 60° based on specific absorption rate limitations, however, the flip angle may provide another parameter for optimization in future studies. FISS is susceptible to B_0_ inhomogeneities and demands, therefore, adequate shimming. It is also conceivable to apply FISS without RF phase cycling in applications with expected large B_0_ inhomogeneities to broaden the pass band at the cost of reduced fat suppression.

Based on a preliminary study (Supporting Information Text S1), an *α*/2 RF pulse was more favorable compared with linear ramp‐up pulses, because of better fat suppression, shorter scan time and reduced RF energy deposition. RF phase cycling between the modules was found to ensure sufficient RF spoiling. This is in agreement with the proposed spoiling in Koktzoglou and Edelman.^17^

The proposed method has several limitations. The periodic interruption of the bSSFP train (including the gradient spoiling) and the dummy prepulses increase scan time, e.g., for *n* = 1 by approximately a factor of 3. In terms of acquisition time, higher FISS module numbers should be favored. Hence, future studies will focus on the investigation of possible trade‐offs between scan time and fat suppression efficiency. This study did not compare FISS with other fat suppression techniques for the in vivo applications. Phantom measurements reveal similar fat suppression efficiency of FISS compared with SPIR, but more thorough in vivo analysis is required. This work focused only on the applicability of FISS to 3D Cartesian imaging. Comparison to the previously proposed 2D radial FISS should be conducted in future studies.

## 5 CONCLUSIONS

Here, we propose an extension of the previously proposed 2D radial FISS sequence to 3D Cartesian which was evaluated in phantom and in vivo measurements for 3 applications: upper thigh angiography, abdominal imaging, and free‐running cardiac CINE. Extensions include RF phase cycling, gradient spoiling, dummy preparation pulses, and appropriate FISS timings. Numerical EPG simulations recommend optimal imaging parameters which were tested and linked to the 3 applications. Overall a good and homogeneous fat suppression was achieved. 3D Cartesian FISS offers an intrinsic fat suppression without the need for dedicated prepulses, which makes it an ideal candidate for fat‐suppressed imaging acquisitions such as 3D peripheral angiography or free‐running 5D whole‐heart CINE.

## Supporting information


**TEXT S1** Comparison to previous work using Cartesian FISS with linear ramp‐up RF pulses instead of *α*/2 ramp‐up RF pulse
**FIGURE S1** EPG simulations: Temporal on‐resonant behavior of bSSFP and FISS acquisitions for changing repetition time TRb and, respectively, imaging resolution. Lines show the transition to steady‐state of fat (blue), blood (black), liver (red) and myocardial (green)
**FIGURE S2** Influence of gradient spoiling and RF phase cycling on spectral behavior (in reached steady‐state, 600^th^ FISS module) of FISS *n* = 1, 2, 4 acquired with 2.1 mm^3^ isotropic resolution, TRb=2.93 ms, TRf = 8.36 / 11.29 / 17.15 ms (*n* = 1, 2, 4), t_spoil_ = 2.5 ms, flip angle = 50°. Lines with the same color (fat (blue), blood (black), liver (red) and myocardial (green)) and different line style (for FISS *n* > 2) show the profiles of each individual bSSFP readout
**FIGURE S3** Representative slice and axial/coronal maximum intensity projections (MIP) for bSSFP and FISS acquisitions (*n* = 1, 2, 4, *α*/2 RF, *D* = 200) in upper thigh of a lean subject with body mass index of 20 kg/m^2^. Vessel depiction is possible with FISS and impaired in bSSFP
**FIGURE S4** Comparison of Cartesian sampling trajectory in upper thigh and abdominal FISS *n* = 1, 2 with *α*/2 ramp‐up pulse and *D* = 200 for 1.8 mm^3^ isotropic resolution
**FIGURE S5** 3D Cartesian FISS pulse sequence diagram showing the continuous acquisition train with dummy prepulses followed by FISS modules with linear r = 5 RF ramp‐up/down pulses. One FISS module (n = 2 here) consists of [+ramp‐up, −α, ADC, +α, ADC, −α, + ramp‐down] with flip angle α and receiver ADC
**FIGURE S6** Phantom measurements to examine the influence of a) ramp‐up RF pulses, b) gradient spoiling and dummy prepulses (for linear r = 10 ramp‐up pulses) over repetition time TRf. Lines show the respective average signal intensity measured in a VOI of an oil phantom which has been imaged with bSSFP (dashed lines), bSSFP with SPIR fat saturation (dash‐dotted lines), GRE (dotted lines) and FISS (continuous lines). Each point along the FISS lines corresponds to a FISS module number n = 1, 2, 3, 4, 5, 10, 15, 20, 25, 30 (from left to right)
**FIGURE S7** Transverse maximum intensity projections in upper thigh for bSSFP and FISS acquisitions (n = 1, 2, 3, 4, 10) with linear r = 5 RF ramp‐up pulses and with (D = 200) and without (D = 0) dummy prepulses acquired with 1.8 mm^3^ isotropic resolution (TRb = 3.06 ms, TRf = 8.62 ms). The spectral behavior depicts the frequency response of fat (blue), blood (black), liver (red) and myocardial (green). Lines with the same color and different line style (for FISS n > 2) show the profiles of each individual bSSFP readout. Dashed vertical line indicates the resonance frequency of fat tissue (3.5 ppm ≡ ±220 Hz for 1.5T)
**VIDEO S1** Cardiac motion‐resolved images at end‐expiration of a free‐running cardiac CINE acquisition at 1.4 mm^3^ isotropic resolution with bSSFP and FISS (*n* = 2, *α*/2 RF, *D* = 200) for whole‐heart coverage. Data was retrospectively binned into 4 respiratory and 8 cardiac phases from an acquisition of 7:52 min:s duration (with an acceleration factor *R* = 3 for FISS), i.e., roughly twice the amount of data is available for bSSFP in comparison to FISS
