# Supplementary material for: 3D Cartesian fast interrupted steady‐state (FISS) imaging
Source: Magn Reson Med. 2019 Jun 14;82(5):1617–30. doi: 10.1002/mrm.27830 (PMC6772102; doi:10.1002/mrm.27830)
Supplement: Supplementary file 1 — TEXT S1 Comparison to previous work using Cartesian FISS with linear ramp‐up RF pulses instead of α/2 ramp‐up RF pulse FIGURE S1 EPG simulations: Temporal on‐resonant behavior of bSSFP and FISS acquisitions for changing repetition time TRb and, respectively, imaging resolution. Lines show the transition to steady‐state of fat (blue), blood (black), liver (red) and myocardial (green) FIGURE S2 Influence of gradient spoiling and RF phase cycling on spectral behavior (in reached steady‐state, 600th FISS module) of FISS n = 1, 2, 4 acquired with 2.1 mm3 isotropic resolution, TRb=2.93 ms, TRf = 8.36 / 11.29 / 17.15 ms (n = 1, 2, 4), tspoil = 2.5 ms, flip angle = 50°. Lines with the same color (fat (blue), blood (black), liver (red) and myocardial (green)) and different line style (for FISS n > 2) show the profiles of each individual bSSFP readout FIGURE S3 Representative slice and axial/coronal maximum intensity projections (MIP) for bSSFP and FISS acquisitions (n = 1, 2, 4, α/2 RF, D = 200) in upper thigh of a lean subject with body mass index of 20 kg/m2. Vessel depiction is possible with FISS and impaired in bSSFP FIGURE S4 Comparison of Cartesian sampling trajectory in upper thigh and abdominal FISS n = 1, 2 with α/2 ramp‐up pulse and D = 200 for 1.8 mm3 isotropic resolution FIGURE S5 3D Cartesian FISS pulse sequence diagram showing the continuous acquisition train with dummy prepulses followed by FISS modules with linear r = 5 RF ramp‐up/down pulses. One FISS module (n = 2 here) consists of [+ramp‐up, −α, ADC, +α, ADC, −α, + ramp‐down] with flip angle α and receiver ADC FIGURE S6 Phantom measurements to examine the influence of a) ramp‐up RF pulses, b) gradient spoiling and dummy prepulses (for linear r = 10 ramp‐up pulses) over repetition time TRf. Lines show the respective average signal intensity measured in a VOI of an oil phantom which has been imaged with bSSFP (dashed lines), bSSFP with SPIR fat saturation (dash‐dotted lines), GRE (dotted lines) and FISS (con [file MRM-82-1617-s001.pdf]

Supporting Information for "Flexible and Efficient Optimization of Quantitative Sequences using Automatic Differentiation of Bloch Simulations" (Lee et al.)

Additional Information on Sequences Optimized using Automatic Differentiation with TensorFlow

In TensorFlow, each operation adds a node to the graph. Since TensorFlow compiles the graph before it can be executed (Abadi, 2016), large graphs incur long compilation times. Adding TR blocks also increases the number of steps for backpropagation. Limiting the number of TRs to the number required to reach steady-state is essential to maintaining reasonable compilation and execution times.

Creating separate nodes for common operations between TRs can be costly, but some operations can be pre-computed and reused. For example, in the DESPOT1 graph, matrices that apply relaxations can be computed once and reapplied at each TR. The RF rotations cannot be reused between TRs due to the quadratic phase variation. The number of nodes for graphs of different sequences are given in Tables S1 and S2.

Although TensorFlow employs GPU parallelization for fast training of neural networks, our work does not benefit from this parallelization since the graphs for the steady-state sequences have many nodes, each with a small amount of computation. However, GPU acceleration could be leveraged when simulating many isochromats. This could apply when simulating slice profiles, a scenario where EPG is limited.

TensorFlow provides a tool (TensorBoard) to visualize computational graphs constructed within the framework. These are useful for debugging and provide a rapid way of plotting a graph. Figures S1, S2, and S4 show the graphs generated for the DESPOT1, DESS, and MRF CRLB calculations.

Supporting Information for "Flexible and Efficient Optimization of Quantitative Sequences using Automatic Differentiation of Bloch Simulations" (Lee et al.)

DESPOT1

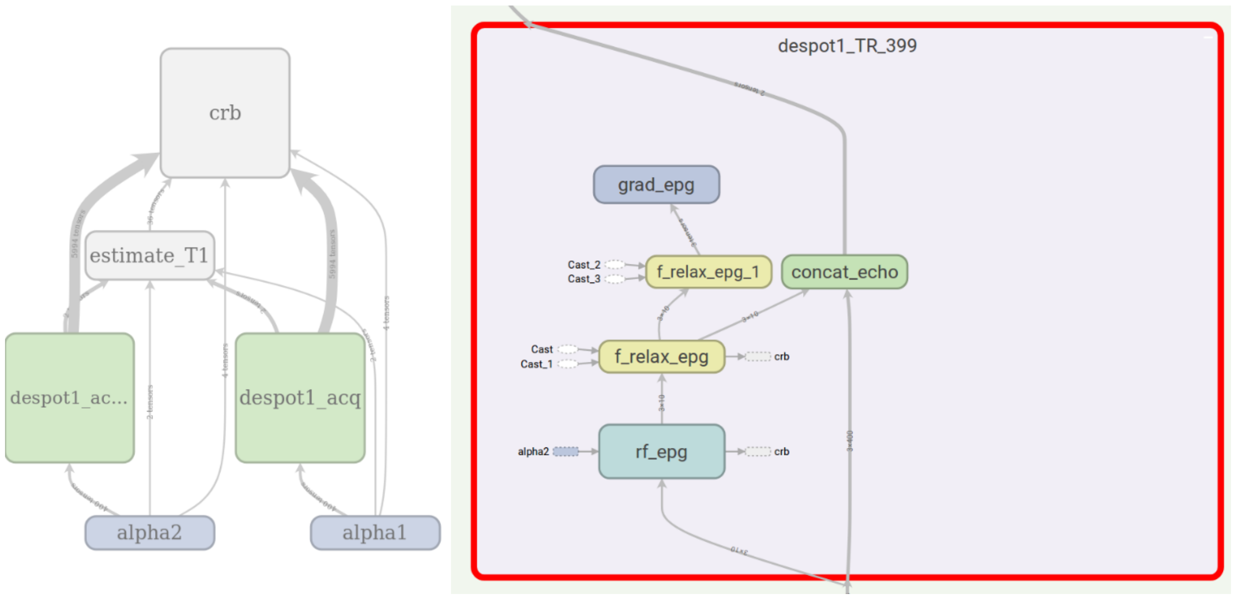

Supporting Information Figure S1: Left: The overall graph structure for DESPOT1. Arrows represent the flow of tensors within the graph. The 'alpha1', and 'alpha2' blocks are scalar tensors that are variable inputs to the graph. Each of the 'despot1_acq' blocks perform the forward Bloch simulation. The 'estimate_T1' block estimates T1 from the linearized steady-state signal and requires inputs from each of the Bloch simulations, as well as the flip angles used to acquire that steady state-signal. The 'crb' block contains nodes that backpropagate through the forward Bloch simulation. All tensors flow to the 'crb' block, since the forward pass is required to compute backpropagation. **Right:** Visualization of the TR in the DESPOT1 simulation. The magnetization tensor undergoes an RF pulse, one relaxation to calculate the echo, one relaxation to reach the next TR, followed by a spoiler gradient. The 'concat_echo' block appends the magnetization at the echo time to a list for debugging purposes.

Double Echo in Steady State (DESS)

We present the CRLB optimization of this sequence in the Supplementary Information to show the flexibility of our method.

Double-Echo in Steady State (DESS) has been used to quickly acquire high resolution T2 maps in the knee (Chaudhari, 2018). DESS captures two images with different contrasts by acquiring two echos in a single TR. The two echos are separated by a spoiler gradient. The first image S1 has an effective echo time of TE, while the second image S2 has an effective echo time of 2TR – TE. The S2 image has reduced SNR due to additional T2 decay and because the primary signal contribution is from magnetization that was rephased after the second RF pulse. The T2 can be estimated from the ratio image S2/S1 if the T1 of the tissue is known using Eq. 7 from Sveinsson et al. (Sveinsson, 2017).

For DESS, we used automatic differentiation to calculate $\left(\frac{\partial S_{i\ DESS}}{\partial\ M_0}\right)$ and $\left(\frac{\partial S_{i\ DESS}}{\partial\ T_2}\right)$. Since cartilage and meniscus have considerable natural variation, we verified our result by performing DESS T2 mapping using the ISMRM/NIST T2 system phantom. We optimized the CRLB over the flip angle alpha and the TR for two different samples in the phantom: (T1/T2 = 608/46 ms), (T1/T2 = 176/11 ms), which are close to the T2 of cartilage (T1/T2 = 1200/ 40 ms), and meniscus (T1/T2 = 1000 / 12 ms) at 3T. Constraints on the sequence (5 < alpha < 50 degrees, TE = 6.5 ms, 21 ms < TR < 40 ms) were chosen to give sufficient time for the RF pulse, readout, and spoiler gradients.

The derivative of the CRLB loss with respect to sequence parameters (α, TR) was also computed using backpropagation. One hundred TRs were used to reach steady-state. The optimization was performed for 10 different initializations over the grid alpha=[10:10:50], TR = [25, 35] with 10 EPG states.

The phantom was scanned at 3T (GE Signa Premier) with scan parameters determined by the CRLB optimization (alpha, TR) and the following acquisition parameters: FOV = 22.5 x 22.5 cm, matrix size = 416 x 416 x 176, in-plane resolution 0.54 x 0.54 mm, slice thickness 1 mm, 48-channel head coil, BW = +/- 31.25 kHz, 2 x 2 ARC undersampling. T1 values for the DESS calculation were obtained using Inversion-Recovery T1 mapping. The standard deviation of the T2 estimates from the two acquisitions were compared to show the effect of the CRLB optimization in different species.

Supporting Information for "Flexible and Efficient Optimization of Quantitative Sequences using Automatic Differentiation of Bloch Simulations" (Lee et al.)

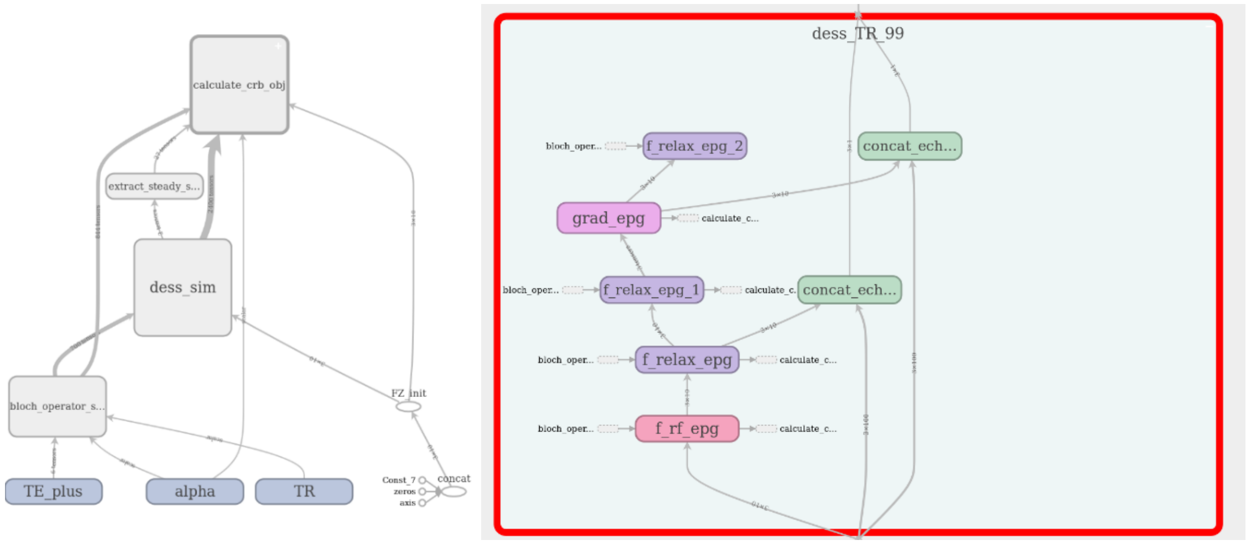

Supporting Information Figure S2: Left: The overall structure of the DESS graph is the same as the DESPOT1 graph shown above. Only one forward Bloch simulation is necessary. The 'bloch_operator' block computes relaxation and RF matrices that can be reused between TRs to reduce the total number of nodes in the graph. **Right:** A single TR Bloch in the DESS simulation. This block has an additional relaxation operation and different ordering compared to DESPOT1 since two echos are acquired in a single TR.

The sequence parameters obtained for the cartilage-like species were alpha = 33 degrees and TR = 32.5 ms. For the meniscus-like species, the parameters were alpha = 45 degrees and TR = 21 ms (minimum). Optimization over 10 different initializations took 357 seconds per tissue on a single CPU. We observed that the solution did not depend on the initialization.

For the sequence targeting the longer T2 species, the TR was lengthened to 32.5 ms to increase the second effective echo time to (2TR – TE) = 58.5 ms. Flip angle differences between the two species take advantage of differing T1 relaxation. The acquisition time was 7:55 minutes for the scan parameters targeting the cartilage-like species, and 5:06 minutes for the meniscus-like species.

The results from the two phantom scans are shown in SI Figure 3. For the cartilage-like species (T2-8), the T2 distribution is Gaussian. For the meniscus-like species (T2-12), the T2 distribution is Rician due to low SNR in the magnitude images.

For vial T2-8, the cartilage-optimized scan improved the T2 standard deviation in the cartilage-like species from 13.4 to 4.9 ms when compared to the meniscus-optimized scan. For vial T2-12, the T2 standard deviation in the meniscus-like species was 0.4 ms when using the meniscus-optimized scan. The T2 estimation for meniscus in the cartilage-optimized scan exhibited considerable mean bias shift. This is caused by low SNR in the meniscus due to T2 decay at the second effective echo time which results in Rician noise bias. The variance was further amplified by Gibbs ringing in the S2 images, which caused T2 estimates larger than 30 ms.

Supporting Information for "Flexible and Efficient Optimization of Quantitative Sequences using Automatic Differentiation of Bloch Simulations" (Lee et al.)

This result demonstrates that the assumption of high SNR made in the ratio distribution approximation must be held for the CRLB optimization to be accurate. Although the cartilage-optimized scan would not be used in practice due to prolonged scan times, poor T2 estimation in the meniscus, and overall reduced SNR efficiency, it demonstrates that the sequence parameters generated with CRLB optimization with automatic differentiation can effectively target different tissues. Using automatic differentiation to optimize the coefficient of variation between the meniscus and cartilage would produce sequence parameters that have more consistent T2 error in each tissue.

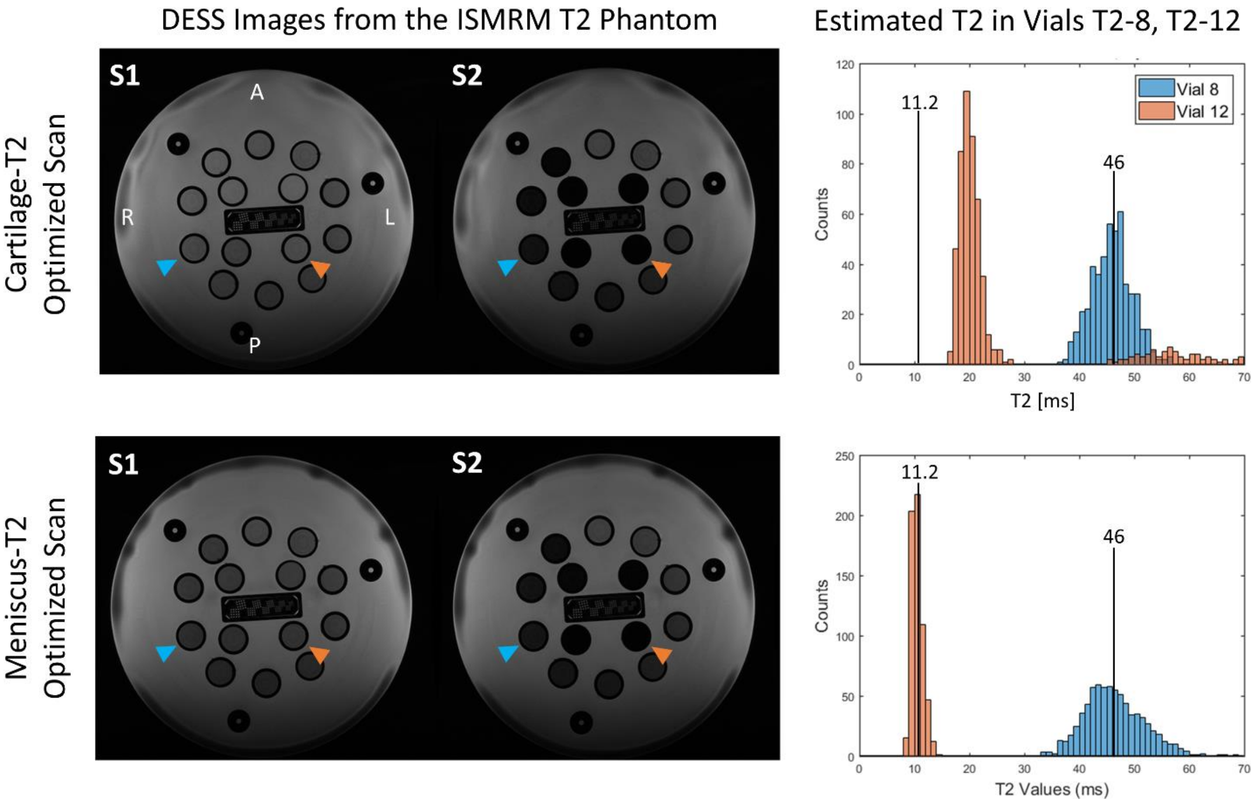

Supporting Information Figure S3: A slice from the ISMRM/NIST T2 phantom is shown. The S2 images have different contrast when compared to the S1 images due to the longer second effective echo time. The histograms for the T2 values estimated in the cartilage-like and meniscus-like vials (indicated by arrows), with the true T2 values denoted by the vertical line, are shown. For the cartilage-like vial, the variance in the cartilage-optimized scan (blue) is reduced compared to the meniscus-optimized scan. For the meniscus-like species, the T2 values (orange) exhibit mean shift and increased variance in the cartilage-optimized scan due to low SNR and Gibbs ringing in the second echo.

Supporting Information for "Flexible and Efficient Optimization of Quantitative Sequences using Automatic Differentiation of Bloch Simulations" (Lee et al.)

Magnetic Resonance Fingerprinting (MRF)

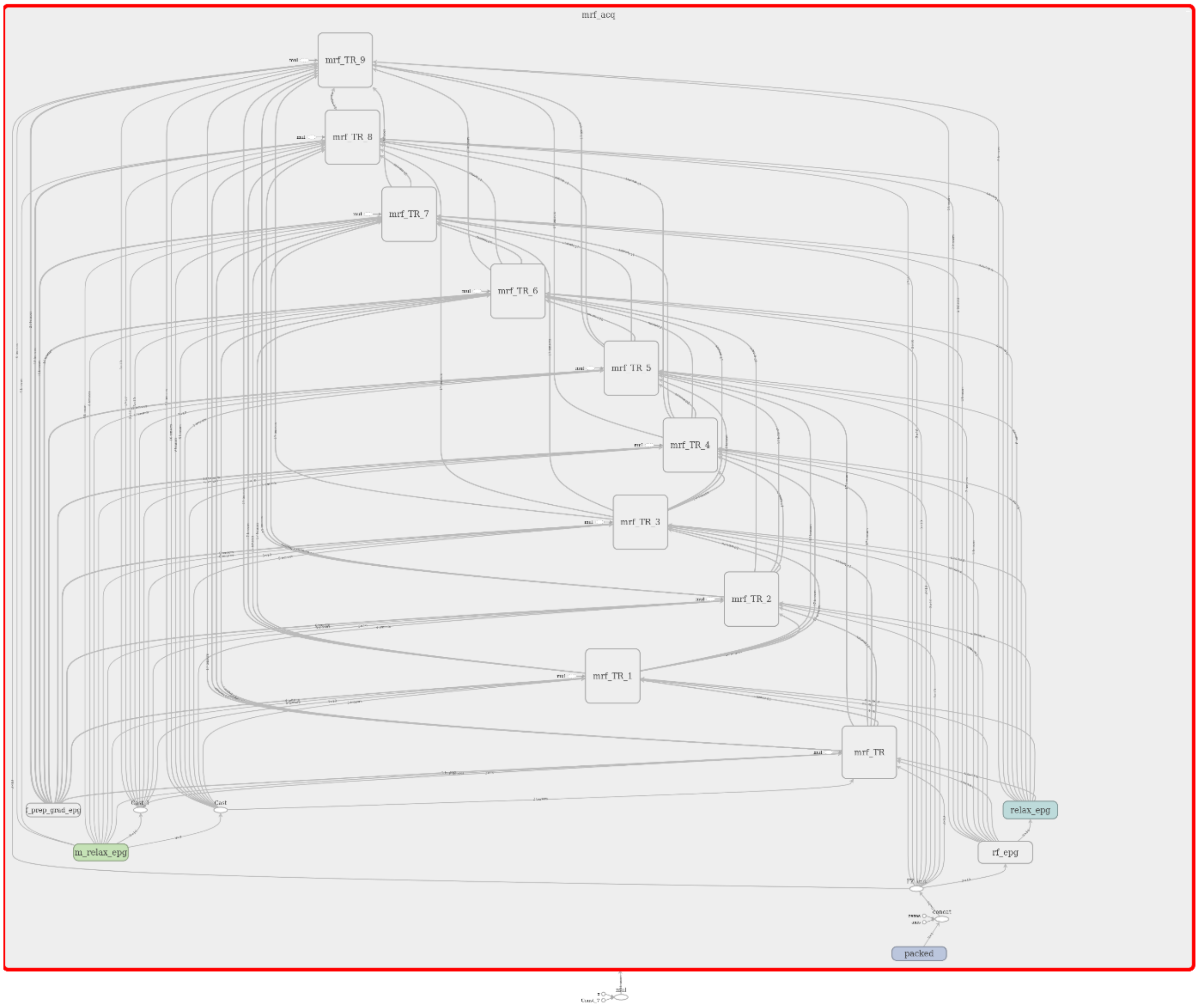

Supporting Information Figure S4: A sample TensorFlow graph for MRF using 10 TRs is shown. This graph demonstrates the quadratic runtime scaling when computing the CRLB using reverse differentiation. For this reason, autograd was used to compute the CRLB for MRF with forward differentiation.

Supporting Information for "Flexible and Efficient Optimization of Quantitative Sequences using Automatic Differentiation of Bloch Simulations" (Lee et al.)

Bloch Operation	Number of Nodes
Relax	20
RF Complex	47
RF Real	23
Spoiler Gradient	13

Supporting Information Table S1: This table shows the number of nodes in a TensorFlow graph for different Bloch operations. The number of nodes affects the compilation and runtime of the graph. For example, applying an arbitrary RF rotation in EPG requires 47 nodes, but this can be reduced to 23 if it is assumed that only real EPG states are used (phi = 90 degrees).

Sequence	Breakdown	Total Number of Nodes
DESPOT1, 2 flip angles<br>400 Repetitions<br>Reusing Relax1 and Relax2	Setup: None<br>Sequence alpha1: 28037<br>Sequence alpha2: 28037<br>CRLB calculation: 58547	114621
DESS, no reuse of operators<br>100 Repetitions	Setup: None<br>Sequence: 13000 (130/ TR)<br>CRLB calculation: 11309<br>dCRLB_wrt TE, alpha, TR: 63154	87463
DESS, reuse of Relax1, Relax2, Relax3, and RF operators<br>100 Repetitions	Setup: 100<br>Sequence: 3000 (30/ TR)<br>CRLB calculation: 6576<br>dCRLB_wrt TE, alpha, TR: 20876	30552

Supporting Information Table S2: This table shows the total number of nodes in the graph for the DESS and DESPOT1 sequences shown in SI Figures 1 and 2. Reusing precomputed matrix operators greatly reduces the number of nodes, and hence the runtime of the graph. Backpropagating through the CRLB calculation in TensorFlow greatly increases the number of nodes in the graph and the memory overhead.

Using Autograd for Non-EPG Bloch Simulations

In this section, we apply automatic differentiation to reproduce one of the optimizations in Reeth et al. (2018). We design a prep pulse with 3 discrete flip angles, and 3 discrete TRs to maximize the contrast between two tissues corresponding to the experiment in Reeth Section 2.7. The gradient of the objective (the contrast) with respect to the control variables (alphas, TRs) is calculated using automatic differentiation. This allows us to perform this optimization without using finite differences. The contrast is calculated for 100 off-resonance values ranging from –500 Hz to 500 Hz, which can easily be simulated. We used the SLSQP implementation with the number of iterations capped to 100.

The contrast was maximized for (T1a, T2a) = 1097, 58, and (T1b, T2b) = 1353, 66 ms

The flip angle was constrained to (-180, 180) degrees, and the TR was bound to (5, 3000) ms. The flip angle was initialized with (90, 180, 90), and the TR was initialized with (58, 58, 1000) ms. The value of beta was 0.0001 which favours a minimum time solution.

The optimization converged in 100 iterations, with the optimal answer being: flip angles (90, 180, 90), and TRs (14, 14, 677) ms. This is similar to the optimal solution obtained by Reeth et al. with small discrepancies in the repetition time due to differences in beta.

Interestingly, the optimization converges to a 'spin echo' solution (180 degree refocusing pulse with equal spacing on either side) which is well known to be robust against off-resonance.

Fisher Information at Different Iterations of MRF CRLB Optimization

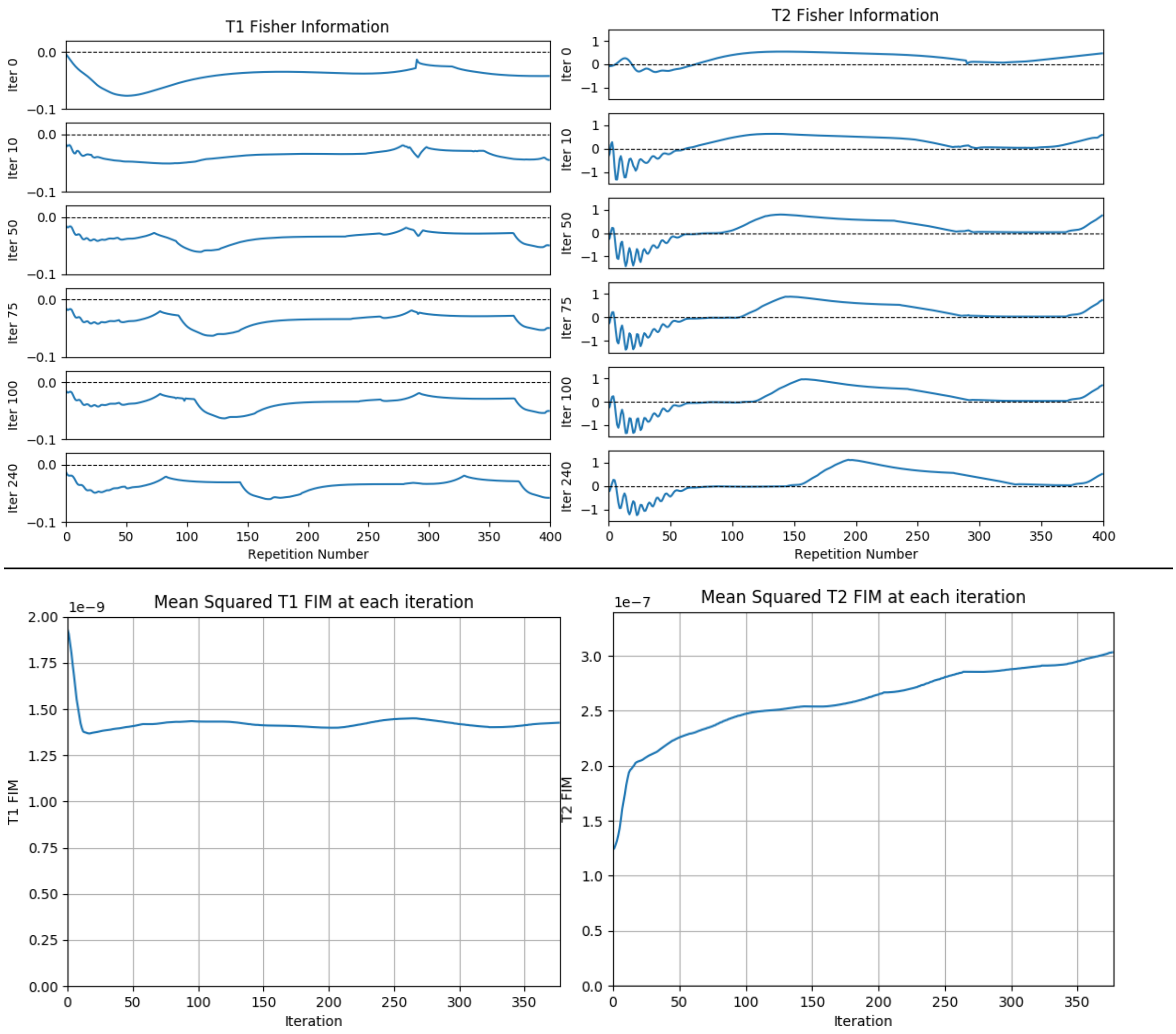

Supporting Information Figure S5: Top: The Fisher Information (FIM) at each echo for different iterations is shown. Although the CRLB depends on the orthogonality of the FIM due to the matrix inversion, a scalar increase in the FIM at each echo will improve (reduce) the CRLB. An interesting observation is that the T2 FIM in the optimal solution is very small between TRs 50 and 150, implying that early echos have very little T2 contrast. **Bottom:** The mean squared T1 and T2 FIM at each iteration of the optimization is shown. The T1 FIM is reduced by a small amount, while the T2 FIM approximately doubled. This is reflective of the improvement in the relative T1 and T2 CRLB components shown in the main manuscript.

Flip Angle and TR Convergence for MRF CRLB SLSQP Optimization

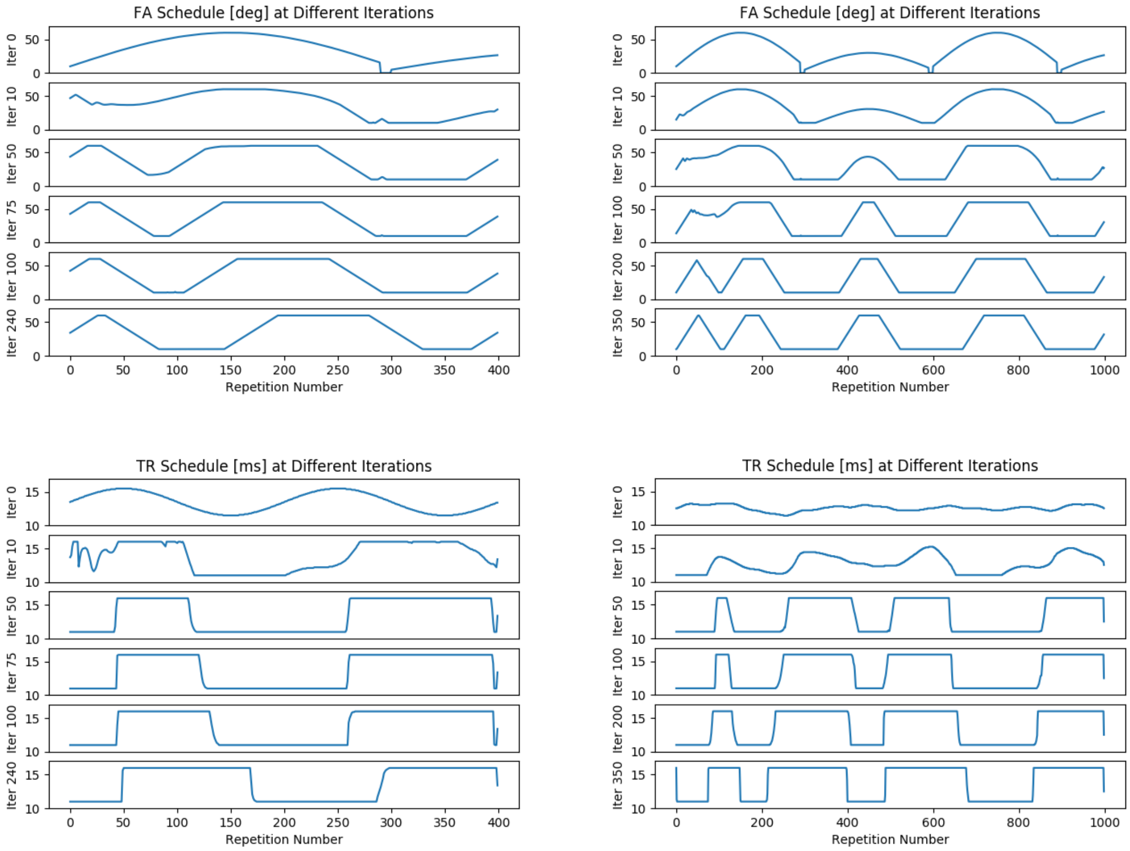

Supporting Information Figure S6: Convergence progression of the SLSQP optimization at different iterations for MRF IR-FISP with 400 TRs (left) and 1000 TRs (right). For the 1000 TR case, the TRs converge at around iteration 100 and cease to update after they have converged. This suggests that a greedy optimization approach may achieve a reasonable solution. A greedy heuristic would lock variables that have converged at the bounds set by the constraint after a minimum number of iterations, reducing the step update time which would improve runtimes for large numbers of parameters. For the 1000 TR case, the optimization took roughly 16 CPU hours for 350 iterations.

Sensitivity of the CRLB Optimized Sequence to B1 and Off-Resonance

To evaluate the bias sensitivity of the CRLB sequence to confounding factors in vivo, we performed a simulated dictionary matching experiment for the CRLB optimized and initialization schedules. An ideal dictionary assuming B1 = 1.0 and on-resonant (df = 0) was generated for tissues T1 = [1200:3:1500], T2 = [50:1:120] ms. We matched a noiseless fingerprint with T1/T2 = 1330/80 ms to this dictionary for B1 values of 0.8 to 1.2, and off-resonance values ranging from –40 Hz to 40 Hz. The results are shown in Figure S7. The estimated T1 and T2 of the conventional sequence is insensitive to off-resonance. The CRLB optimized sequence T2 estimate becomes <u>shorter</u> and the T1 estimate becomes <u>longer</u> as the off-resonance increases. The simulation also indicates that the CRLB optimized sequence has increased sensitivity to B1.

Dictionary matching is unbiased assuming that the model is ideal. Unmodelled phenomena skews the parameter match to certain T1/T2 values. As an extreme case, suppose that a strong water fingerprint was erroneously added to each pixel in the time series. If unaccounted for in the dictionary, the dictionary match would result in all voxels being water. This is the principle used in multicomponent fingerprint matching, where multiple T1/T2 fingerprints are identified per voxel (McGivney, 2018) and the strongest T1/T2 fingerprint shows the strongest cluster.

The bias from this simulation does not exactly match the bias that is observed in vivo but demonstrates how confounding factors can have a greater effect on the CRLB optimized sequence. In vivo, we observe a <u>longer</u> T2 and a <u>longer</u> T1 when using the CRLB optimized sequence. This simulation matches the bias trend that exists in MRF phantom experiments shown in Figure S8.

In Figure S8, we performed ten consecutive undersampled MRF acquisitions of the ISMRM NIST T2 phantom for each of the 1000 TR CRLB optimized and conventional sequence, using the schedule shown in Figure S6. The reference values are published T1/T2 values for the phantom obtained using NMR (Keenan, ISMRM 2016, Program #3290). The scan was done at 3T using an 8-channel head coil and each scan was approximately 14 seconds. The FOV was 22.5 cm, with a matrix size of 192 x 192. The magnetization was allowed to fully recover to equilibrium between acquisitions. Calculating the mean T1 and T2 in each sample ROI allows us to quantify the bias, and the normalized standard deviation across separate acquisitions demonstrates the improvement of the CRLB optimized sequence against white noise. The normalized standard deviation was computed for each voxel in the phantom by calculating the standard deviation of the T1/T2 estimate in the acquisitions dimension and dividing by the mean T1/T2 estimate of that voxel. Figure S8 shows that the CRLB sequence has improved performance against white noise and that bias in the T1/T2 estimate exists after changing the sequence parameters.

We also acquired three different slices from the brain of a consenting volunteer under IRB approval using a 1000 TR conventional and CRLB optimized sequence. The scan was performed using undersampled spirals and reconstructed using dictionary matching. T2 maps from the acquisition are shown in Figure S9 and demonstrate that the in vivo T2 bias trend observed in the fully sampled scan also exists in the undersampled scans.

Supporting Information for "Flexible and Efficient Optimization of Quantitative Sequences using Automatic Differentiation of Bloch Simulations" (Lee et al.)

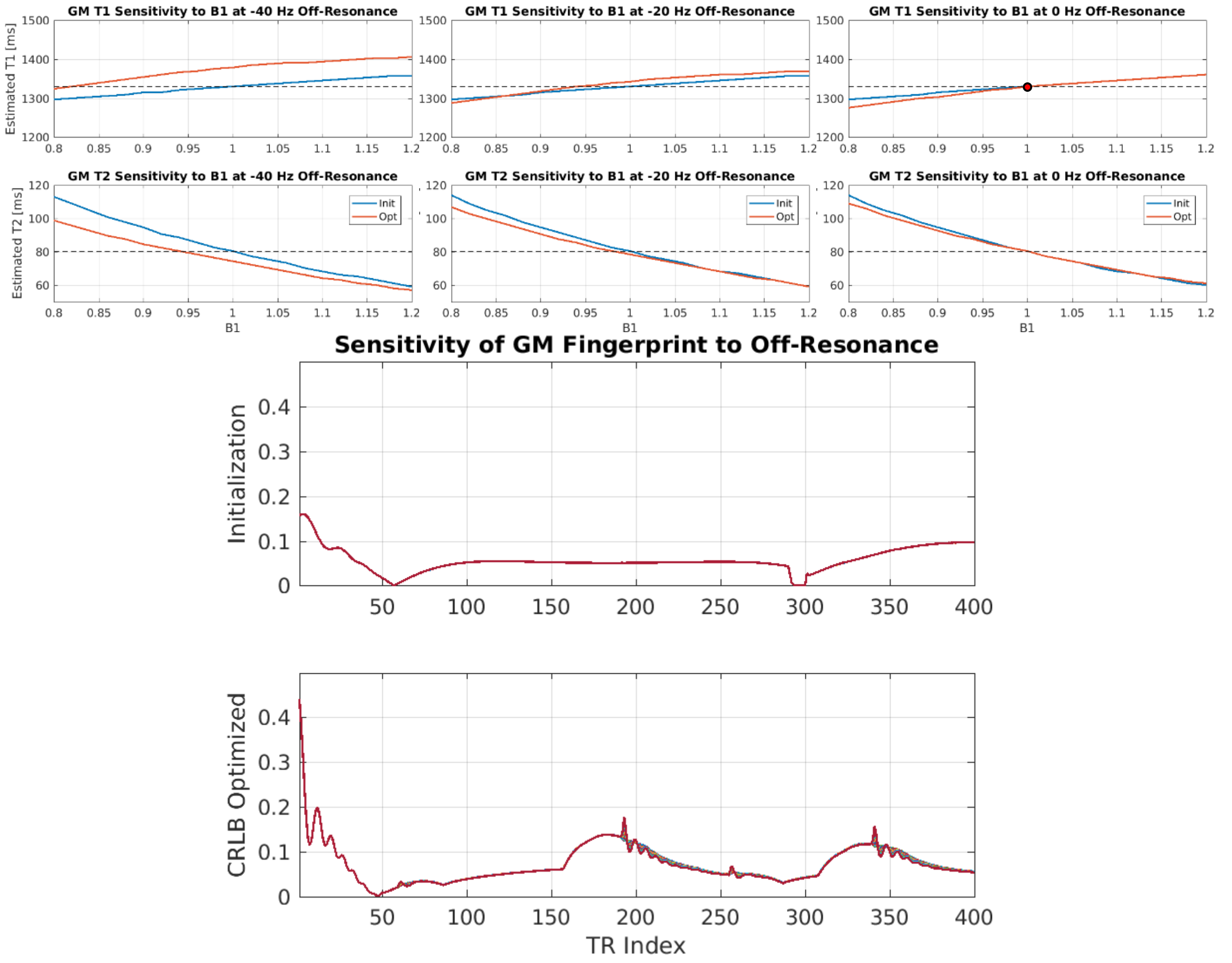

Supporting Information Figure S7: Top: The result of the imperfect fingerprint matched to the ideal dictionary demonstrates greater sensitivity of the CRLB optimized fingerprint to off-resonance and B1 effects. The conventional schedule has little sensitivity to off-resonance. The true value of the tissue of T1/T2 = 1330/80 ms is marked as the dashed black line. The on-resonance, no B1 variation simulation is marked with a circle and shows no bias. **Bottom:** Conventional and CRLB optimized fingerprints for different values of off-resonance. In the conventional sequence, there is no visible deviation of the fingerprint. In the CRLB optimized sequence, deviations are apparent at TR index 200 and 350.

Supporting Information for “Flexible and Efficient Optimization of Quantitative Sequences using Automatic Differentiation of Bloch Simulations” (Lee et al.)

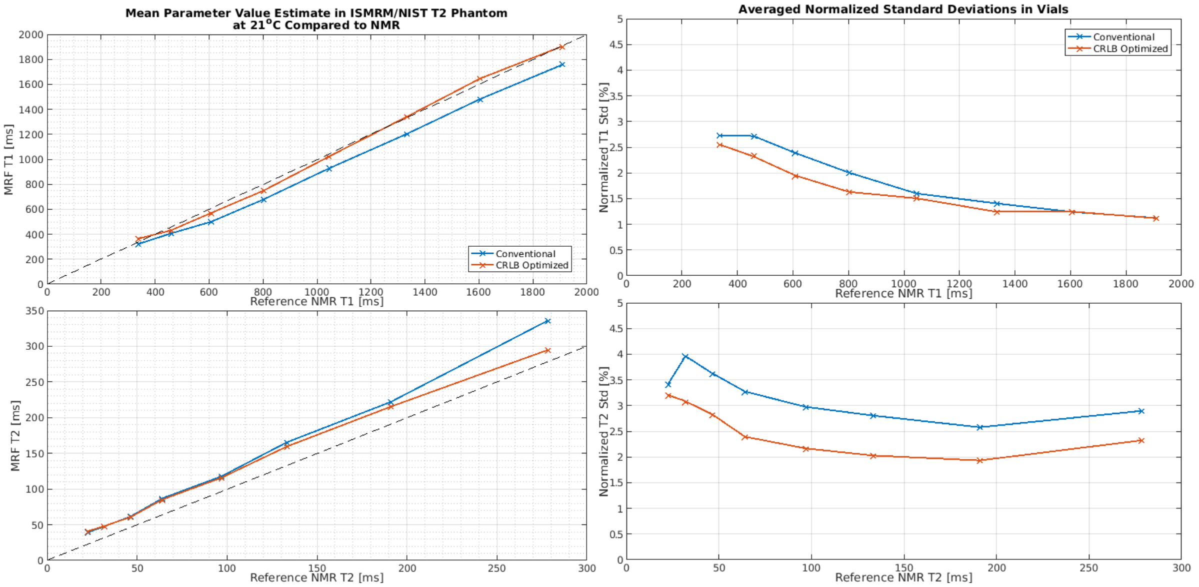

Supporting Information Figure S8: Left: Mean parameter estimates obtained in the ISMRM NIST T2 phantom are compared for the CRLB optimized (red) and conventional (blue) MRF schedules. Reference T1 and T2 values on the x-axis are published parameter values obtained from NMR. The CRLB Optimized schedule overestimates T1 across the range of sample values relative to the conventional schedule. The T2 estimate from the CRLB optimized schedule is marginally underestimated compared to the conventional schedule for the 100 - 200ms range, which is opposite to what is observed in vivo. **Right:** The mean normalized standard deviation across each sample for the repeated undersampled acquisition is shown. Similar to the fully sampled in vivo measurements, the performance of the T1 estimate is unchanged. The error in the T2 estimate is improved across the samples, but the magnitude of the improvement is reduced compared to the fully sampled in vivo case. This may be due to increased off-resonance in the ISMRM NIST phantom, since the samples are small and have a plastic shell to separate it from water.

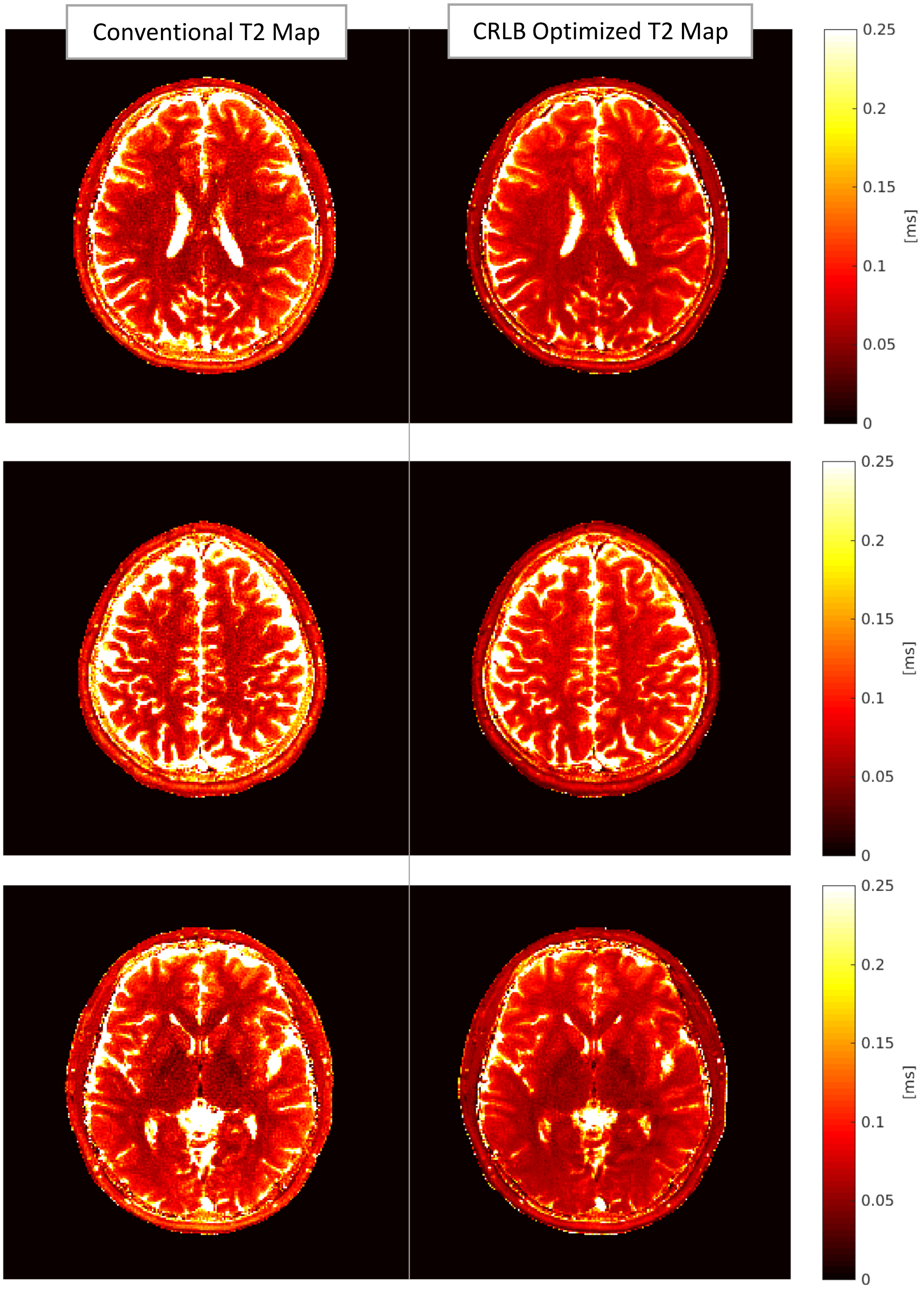

Supporting Information Figure S9: T2 maps from the 1000 TR undersampled conventional acquisition and CRLB optimized acquisitions are shown in the left and right columns respectively. T2 maps from undersampled in vivo scans exhibit the same bias trend as the fully sampled in vivo scan where T2 is overestimated when using the CRLB optimized acquisition. Visually, the CRLB optimized acquisition provides T2 maps that are more uniform with less noise.

Asymptotic Runtime of Autograd for MRF CRLB Calculation

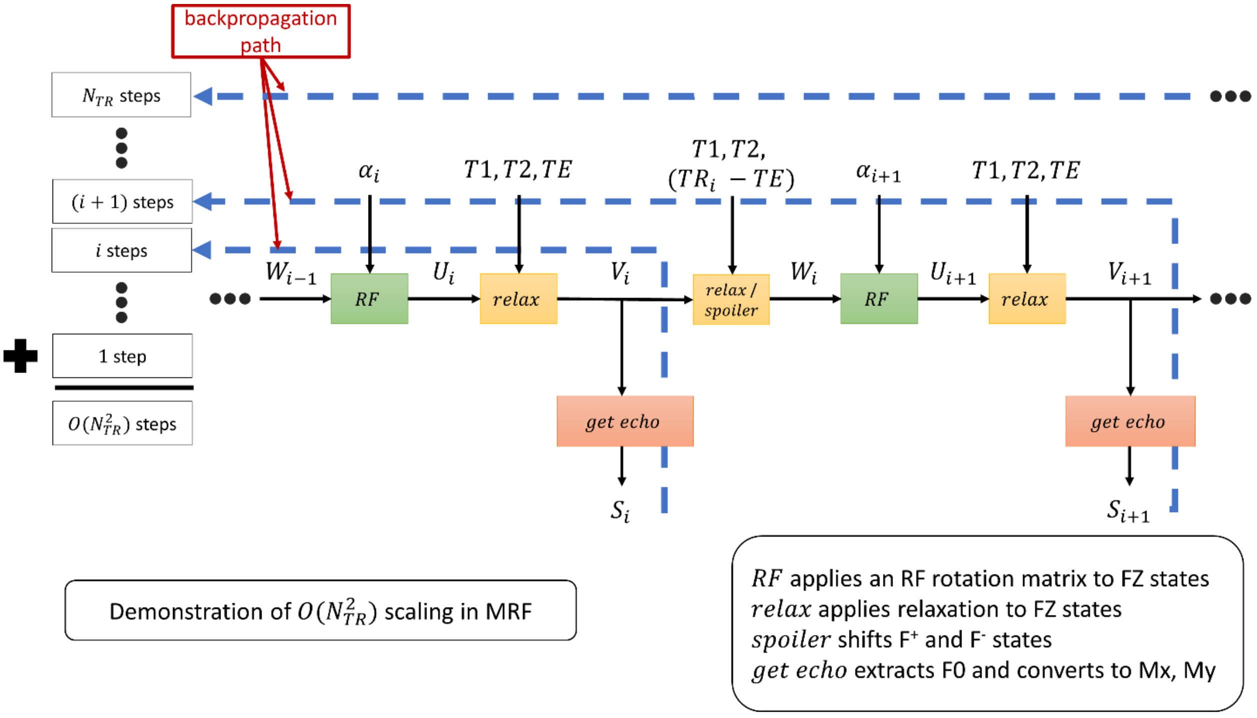

Supporting Information Figure S10: The computation graph for the MRF sequence and the backpropagation steps required to calculate the CRLB is shown. Since reverse mode differentiation propagates to the first node of the graph, TR_K must backpropagate through K TR blocks. The total number of operations to compute the CRLB is therefore $\sum_{i=1}^{N_{TR}} i = O(N_{TR}^2)$. In MRF, the number of parameters in the optimization also scales with $O(N_{TR})$. If the gradient of the CRLB is approximated using finite differences, this results in $O(N_{TR}^3)$ runtime scaling for a single evaluation of the objective and gradient. Using forward automatic differentiation to calculate the CRLB reduces its runtime to $O(N_{TR})$ resulting in $O(N_{TR}^2)$ runtime scaling for a single evaluation of the objective and gradient when using finite differences. Calculating the gradient of the CRLB with respect to sequence parameters using backpropagation further reduces the asymptotic runtime of the gradient and CRLB calculation to $O(N_{TR})$.
